# CENP-A and H3 Nucleosomes Display a Similar Stability to Force-Mediated Disassembly

**DOI:** 10.1371/journal.pone.0165078

**Published:** 2016-11-07

**Authors:** Sung Hyun Kim, Rifka Vlijm, Jaco van der Torre, Yamini Dalal, Cees Dekker

**Affiliations:** 1 Department of Bionanoscience, Kavli Institute of Nanoscience Delft, Delft University of Technology, Delft, the Netherlands; 2 Chromatin Structure and Epigenetic Mechanisms Unit, Laboratory of Receptor Biology and Gene Expression, Center for Cancer Research, National Cancer Institute, NIH, Bethesda, Maryland, United States of America; Northeastern University, UNITED STATES

## Abstract

Centromere-specific nucleosomes are a central feature of the kinetochore complex during mitosis, in which microtubules exert pulling and pushing forces upon the centromere. CENP-A nucleosomes have been assumed to be structurally unique, thereby providing resilience under tension relative to their H3 canonical counterparts. Here, we directly test this hypothesis by subjecting CENP-A and H3 octameric nucleosomes, assembled on random or on centromeric DNA sequences, to varying amounts of applied force by using single-molecule magnetic tweezers. We monitor individual disassembly events of CENP-A and H3 nucleosomes. Regardless of the DNA sequence, the force-mediated disassembly experiments for CENP-A and H3 nucleosomes demonstrate similar rupture forces, life time residency and disassembly steps. From these experiments, we conclude that CENP-A does not, by itself, contribute unique structural features to the nucleosome that lead to a significant resistance against force-mediated disruption. The data present insights into the mechanistic basis for how CENP-A nucleosomes might contribute to the structural foundation of the centromere *in vivo*.

## Introduction

Nucleosomes undergo various types of stresses *in vivo* [1, 2]. Transcription and replication machineries, chromatin remodelers, and DNA repair machinery all require eviction or remodeling of nucleosomes in order for these vital processes to occur in eukaryotes [3–5]. During mitosis, centromere-specific nucleosomes endure pushing and pulling forces, despite which they must remain associated with the centromeric DNA for successful chromosome segregation [6]. While the precise force transmitted to each centromeric nucleosome has not been measured *in vivo*, the force mediated by a single microtubule connected to the kinetochore has been estimated to be ~50 pN/microtubule for the point centromeres of budding yeast, whereas 2–8 microtubules are bound to each chromosome in animal cells [7–9]. Consequently, significant forces act on the centromere during mitosis. It is therefore of interest to investigate whether CENP-A nucleosomes, which substitute H3 nucleosomes and serve as a primary epigenetic mark at active centromeres, are structurally unique in their ability to withstand force-induced eviction [10].

In recent years, diverse lines of investigation have started to study this issue. First, *in vitro* work using optical tweezers experiments showed that yeast CENP-A nucleosomes assembled on yeast centromeric or plasmid DNA were unstable, and could quite easily be evicted during application of tension [11]. Because budding yeast centromeres contain a unique 120bp genetic CDE element that directly binds kinetochore proteins [12], it was unclear whether these yeast results would translate to other eukaryotes in which CENP-A serves as the primary epigenetic mark at the centromere. However, recent computational modeling studies support the view that overall, human octameric CENP-A nucleosomes are more flexible than previously suspected [13]. In addition, recent FRET-based *in vitro* assays showed that, in the absence of kinetochore partners, CENP-A nucleosomes have an enhanced pliability [14, 15]. Second, provocative *in vivo* studies have shown that as long as key inner kinetochore proteins are directly tethered to the DNA at an exogenously tagged locus, mammalian CENP-A is dispensable for centromere function [16]. Third, crystallographic and AFM analysis [17–20] suggest that when measured in a static state, CENP-A nucleosomes are very similar to canonical H3 nucleosomes. In contrast to these reports, other experiments have however suggested that octameric CENP-A nucleosomes are more compacted and rigidified *in vitro* [21, 22], or possess alternative conformations *in vivo* [23, 24]. These data support the interpretation that CENP-A might encode a distinctive structural identity, and integrity to its nucleosomes. Here, we directly test whether mammalian CENP-A confers special mechanical properties upon its octameric nucleosome, either on random or centromeric DNA sequences, which could potentially allow it to resist force-induced eviction.

The canonical nucleosomes consist of an octamer of two copies of H2A, H2B, H3 and H4 histones around which 147 bp of DNA is wrapped [25]. *In vitro*, nucleosomes can be formed by salt dialysis [26] or by the use of histone chaperones [27–30], both of which assemble nucleosomes in a quasi-orderly fashion, starting with a tetramer of H3/H4, and the addition of 2 dimers of H2A/H2B upon linear or supercoiled circular DNA templates [31]. For linear DNA molecules held under tension, the end-to-end length decreases upon formation of nucleosomes. Disassembly of nucleosomes under a large force applied on the DNA has been studied extensively [10, 15, 32, 33]. The increase in length upon disassembly has been determined rigorously, allowing for precise measurement of the individual disassembly reaction of a single nucleosome [11, 34, 35]. Under a tension applied along the DNA, a nucleosome loses contact with its DNA in a well-defined two-step process: outer-turn and inner-turn disruption [32, 35, 36]. First, the outer turn of a nucleosome unwraps at a relatively low force in the range of 3–10 pN [35], which is important in the plasticity of the nucleosome structure which can change depending on the supercoiling state of the DNA [37, 38], and for processes such as RNA-polymerase progression along the chromatin fiber, or competition with transcription factors in the site-exposure model [39]. This outer-turn disruption is a reversible process such that the histone proteins remain on the DNA and re-form the intact nucleosome when the applied force is lowered [35, 40, 41]. In contrast, in the second step, an inner-turn disruption occurs upon pulling the DNA with a force in the range of 15–40 pN. This second, irreversible, step directly relates to the stability of the nucleosome [35]. Intrinsic strength of the inner turn, which relies solely on H3-H4 (or CENP-A/H4) tetramer interactions with the DNA, is likely critical for processes like mitosis in which nucleosomes must be retained on the DNA. Consequently, we direct our attention to the inner-turn disruption (i.e. the irreversible nucleosome disassembly) of CENP-A and H3 nucleosomes.

Using magnetic tweezers, we monitored real-time changes in the DNA end-to-end distance at high resolution, during chaperone-assisted assembly and force-mediated disassembly of H3 and CENP-A nucleosomes on varying DNA sequences, including human centromeric DNA [42]. We report that CENP-A and H3 nucleosomes display very similar disassembly curves under high forces that unpeel the inner core of the octameric nucleosomes, and which lead to full disassembly of the histones from the DNA. Surprisingly, even on human centromeric DNA sequences, CENP-A displays only a subtle increase in stability relative to H3. We discuss implications of our data that challenge the notion that CENP-A would have unique structural properties which help it resist force-induced eviction.

## Material and Methods

### DNA

A 7.9 kb-long random-sequence DNA molecule lacking any nucleosome-positioning sequences nor alpha-satellite DNA sequence was made by taking a fragment of the pBlueScript-1,2,4+pSfv1 plasmid, which consists of fragments of Lambda DNA and a fragment from pSfv1 (Invitrogen) in pBluescript SK+ (Agilent). Alpha satellite DNA sequence plasmids were obtained as described in [43]. From this plasmid, DNA fragments each containing 5 alpha satellite sequence repeats were taken and self-ligated to each other to make a final construct of 3.4 kb DNA as a second substrate. For surface immobilization, a 500 bp-long DNA fragment labeled with multiple Dibenzocyclooctyne (DBCO) molecules was ligated at one end of the DNA molecules, while the other end was ligated with a 500 bp-long DNA fragment labeled with multiple biotin molecules for magnetic-bead attachment. In the experiments, we selected, by measuring their torque-responses, rotationally unconstrained DNA molecules that can freely rotate due to the presence of a nick. The complete DNA sequences are given in **S1 Text**.

### Proteins

Recombinant CENP-A, H3, H4 histones (*Homo sapiens*) and H2A and H2B histones (*Xenopus Laevis)* were purified according to the protocol from Luger et al. [44] with modifications by Walkiewicz et al. [45]. Recombinant NAP1 was a kind gift from Alexandra Lusser.

### Magnetic tweezers assay

The details of the magnetic tweezers setup were described in a previous report [46]. In brief, a flow cell was made by sandwiching two cover glass slides with parafilm as a spacer (**S1 Fig**). The bottom cover glass surface was pre-treated with polyethyleneglycol (PEG) functionalized with azide reactive group (PG2-AZSL-5k, Nanocs). The flow cell was then connected with a syringe pump for buffer exchange and placed on the magnetic tweezer setup. The image area of the flow cell was illuminated by a LED source (660 nm, Thorlabs) and the magnetic beads were imaged by using an objective lens (50x, oil immersion, Nikon) and a CCD camera (Falcon 4 M60, Dalsa) operating at a frame rate of 50 Hz. A pair of magnets was placed above the flow cell with a motorized stage such that the magnetic force applied to the beads on the slide glass surface can be controlled by changing the height of the magnets from the surface. The magnet height dependence on the applied tension was calibrated by measuring Brownian fluctuation of the beads held at each magnet height. To determine the z-position of the surface, we measured the bead height at a low force <0.01 pN for 10~20 s at a sampling rate of 50 Hz and used the minimum z-position value within the time window as a z-offset. We are aware that the 10–20 s measurement may not be long enough to determine the z-position accurately; however, our analysis in this study solely depends on the relative changes in the bead height.

DNA molecules were first incubated with magnetic beads (Dynabeads M270, Invitrogen) for 15 min in buffer-A (50 mM TrisHCl pH7.5, 200 mM NaCl, 5mM EDTA, 0.25% (v/v) Tween20). The magnetic beads were then precipitated by using a magnet and washed to remove unbound DNA molecules. The washed beads were reconstituted in buffer-A and flowed into the flow cell followed by 1 hr incubation. To remove unbound magnetic beads, excess amount of buffer-A was flowed into the flow cell. Finally, the buffer was exchanged by the measurement buffer (25 mM Hepes pH 7.6, 50 mM KCl, 0.1 mM EDTA, 0.038% (v/v) polyethylene glycol (PEG), 0.038% polyvinyl alcohol (PVOH).

### Nucleosome assembly

Details of the NAP1-assisted nucleosome assembly are described in our previous report and the references therein [27–30, 37, 47]. For canonical H3 nucleosome assembly, a mixture of 184 nM H3, 184nM H4, 484 nM H2A, 484nM H2B, and 621 nM NAP1 in a buffer carrying 50 mM KCl, 25 mM Hepes pH 7.6, 0.1 mM EDTA, 0.25% PEG, 0.25% PVOH and 1 mg/ml BSA were incubated on ice for 30 min. For CENP-A nucleosomes, 105 nM CENP-A, 105 nM H4, 655 nM H2A, 655nM H2B, and 274 nM NAP1 were used. We added higher molar concentrations of H2A and H2B over CENP-A/H3 and H4 to ensure complete nucleosome assembly. Immediately before the protein mixture was released into the flow cell for nucleosome assembly on the surface-bound DNA, proteins were diluted by 500–1000 folds in the measurement buffer carrying 25 mM HEPES pH7.6, 50 mM KCl, 0.1 mM EDTA, 0.038% (v/v) PEG, 0.038% (v/v) PVOH. PEG and PVOH were added as crowding agents to promote nucleosome assembly. During assembly the tension across the tethered DNA was kept above 0.5 pN to minimize loop formation via inter-nucleosome interactions or via non-specific sticking of the bead on the surface.

### Micrococcal Nuclease digestion assay

Micrococcal Nuclease (MNase) was purchased from sigma (N3755, Sigma). For H3 nucleosome assembly, we first pre-incubated 355 nM H3, 355nM H4, 581 nM H2A, 581nM H2B, and 559 nM NAP-1 in a buffer carrying 50 mM KCl, 25 mM Hepes pH 7.6, 0.1 mM EDTA, 0.25% PEG, 0.25% PVOH and 1 mg/ml BSA on ice. For CENP-A nucleosome assembly we pre-incubated 309 nM CENP-A, 309 nM H4, 484 nM H2A, 484 nM H2B, and 559 nM NAP-1. Then, the protein mixtures were diluted 200 folds immediately before adding 375ng of 2.7 kb DNA (a fragment of randDNA), followed by 15 min incubation. Then, the histone/DNA mixtures were equilibrated at 37°C for 5 min before adding 0.001 unit of MNase and 2 mM of CaCl_2_. The samples were then digested at 37°C for 0, 3 or 10 min, respectively. The reaction is stopped by adding 4 mM of EDTA. After the digestion, we added 0.8 units of Proteinase K (P8107, NEB) and incubate 1 hr at 37°C to remove proteins from the samples. The samples were then mixed with the same volume of Phenol/Chloroform/Isoamylalcohol (P2069, Sigma), followed by spin down at 12000 RPM (13,300g). After the spin down, the supernatants were taken and the DNA fragments were reconstituted after ethanol precipitation. The size of digested DNA molecules were then checked with 1.5% TBE-agarose gel running at 100V for 1.5 hr.

## Results

### NAP1-assisted nucleosome assembly monitored by decrease in DNA end-to-end length

**Fig 1:**
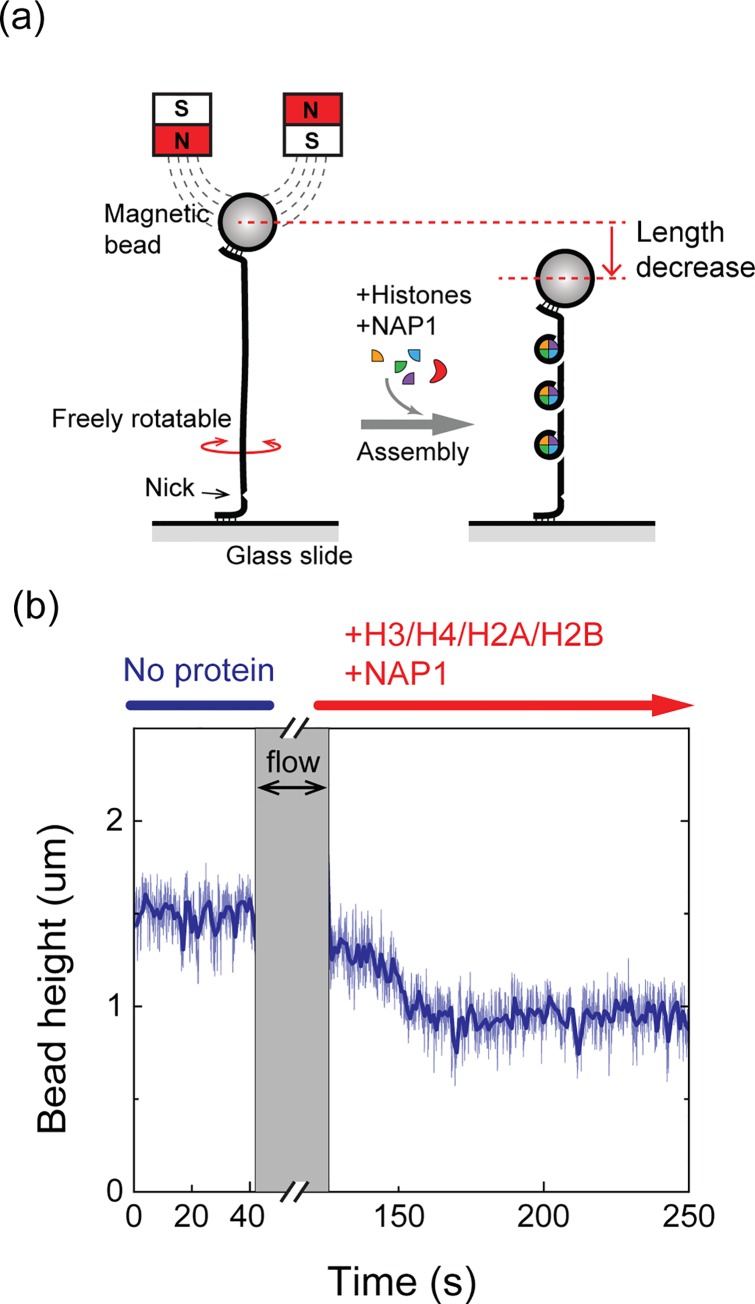
NAP1-assisted nucleosome assembly (a) Schematic diagram of a tethered DNA molecule under tension in the magnetic tweezers assay. Torsionally unconstrained (nicked) DNA molecules were selected for nucleosome assembly. Assembly of a nucleosome results in shortening of the end-to-end length of the DNA. (b) Real-time observation of nucleosome assembly at 0.5 pN. Thin blue line shows raw data at 50 Hz bandwidth and thick blue line is the moving average with 1 s time window. During the flow of a buffer carrying histones and NAP1, the force was increased to 12 pN to suppress non-specific binding of the bead to the surface. After the flow, the force was lowered again to 0.5 pN and shortening of the DNA lengths was observed as nucleosomes assemble.

We assembled nucleosomes on two types of DNA. First, we prepared a 3.5 kb DNA carrying 20 repeats of the consensus alpha satellite centromeric DNA sequence (hereafter, centromeric DNA, “cenDNA”, see **S1 Text** for full sequence) [43, 48]. For comparison, we studied nucleosomes on a 7.9 kb DNA molecule with random sequence (hereafter random DNA, “randDNA”), lacking any alpha satellite DNA sequence. Both DNA samples were additionally ligated at one end to a 500 bp DNA fragment with embedded biotins, and at the other end to a 500 bp fragment with Dibenzocyclooctyl (DBCO). The biotin labeling permits attachment of the DNA to a streptavidin-coated magnetic bead (2.7 μm diameter), while the DBCO labeled-end provides a covalent link to an azide-functionalized glass slide surface via copper-free click chemistry (**Fig 1A**) [46]. The surface-tethered DNA molecules were then put under a pair of magnets, such that the magnetic force exerted on the DNA through the attached magnetic bead can precisely be controlled by changing the height of the magnets to the surface of the slide (**Figs 1 and S1**). The end-to-end length of the stretched DNA was measured by monitoring the diffraction pattern changes of the magnetic bead image [49, 50]. Before each experiment, we checked the torsional response of each DNA by rotating the magnets and we chose only torsionally unconstrained (nicked) DNA molecules to avoid any complexities possibly caused by DNA supercoiling [41, 47].

We assembled nucleosomes containing all 4 histones (H3/CENP-A, H4, H2A, and H2B) with the ubiquitous histone chaperone NAP1 as described in our previous study [47]. Shown in **Fig 1B** is a typical nucleosome assembly trace measured on the random DNA. In a histone-free buffer, the height of the bead was 1.5 μm above the surface, at low (0.5 pN) applied force. We then introduced a pre-incubated mixture of histones and NAP1 into the sample chamber. Since the focus of this study was to test the stability of mononucleosomes, the final protein concentration was empirically chosen such that the number of nucleosomes assembled on a single DNA molecule was low, i.e. less than 10–20 (5–10 for cenDNA), in order to minimize inter-nucleosome interaction. We note that the differences in the lengths of the two DNAs (7.9 kb for randDNA and 3.4 kb for cenDNA) result in a roughly similar density of 1 assembled nucleosome per 400–800 bp on each DNA molecule. Furthermore, magnetic tweezers are force clamps where disruption of a nucleosome does not affect the force on the DNA molecule, making each disassembly event independent of subsequent nucleosome ruptures. During the flowing in of histones, the force was increased to ~12 pN to avoid non-specific sticking of the bead to the surface. After flowing in the proteins, the force was lowered back to 0.5 pN and nucleosome assembly was monitored. A gradual decrease in the bead height with time is notable in the time traces (**Fig 1B**) as nucleosome assembled, inducing compaction of the DNA via the 1.7 turn wrapping around the histone octameric core. When histone mixtures lacking H3 and H4 were flown into the cell, we did not observe any shortening on the DNA length (**S2A and S2B Fig**). To check if the assembled particles in our buffer condition were indeed in the octameric form, we performed MNase digestion assay. Consistent with the crystallographic structures [19], we observed a ~150bp band of the protected DNA region by the octameric form of nucleosomes for H3 nucleosomes and a ~120bp band for octameric CENP-A nucleosomes (**S2C Fig**).

### Both H3 and CENP-A nucleosomes disassemble at similar forces exerted on DNA

**Fig 2:**
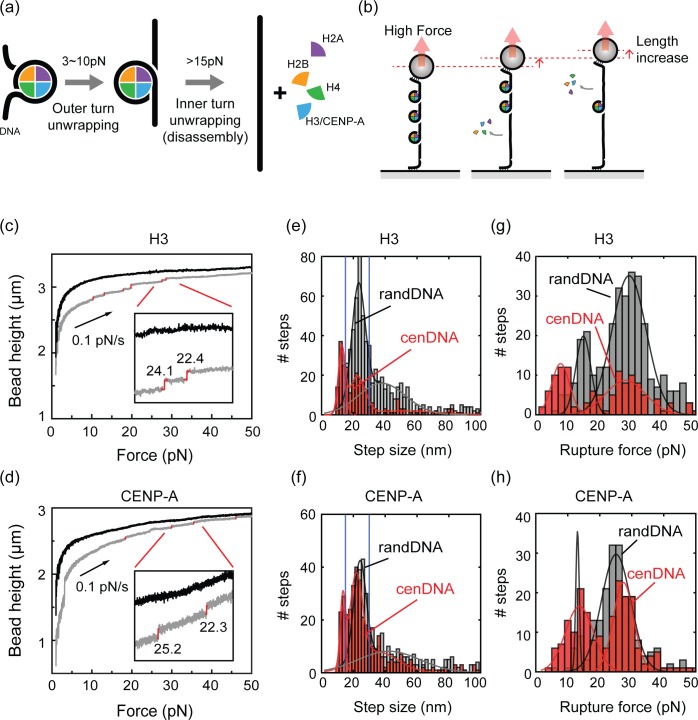
Real-time observation of nucleosome disassembly (a) Model for the two-step process of the nucleosome disruption. Both outer- and inner-turn unwrapping processes release about 20–25 nm of the bound DNA. (b) A schematic diagram of nucleosome disassembly. Unwrapping of the DNA from a nucleosome results in a stepwise increase in the DNA length. (c) Representative force-extension curves measured with random DNA sequence before (black) and after (grey) the H3 nucleosome assembly. The force-extension curves were measured from 1 pN to 50 pN at a constant speed of 0.1 pN/s. After the assembly, multiple stepwise increases of the DNA extension were observed. Step sizes (in nm) determined from a step-finding algorithm indicated with a red vertical line next to each event highlighted. Inset: Magnification of a small region showing two discrete steps. (d) Representative force-extension curves measured with random DNA before (black) and after (grey) the CENP-A nucleosome assembly. (e-f) Step-size distributions of (e) H3 and (f) CENP-A nucleosome disassembly from random DNA (grey bars) and centromeric DNA (red bars). Blue lines indicate the 15–30 nm range expected for single-nucleosomal DNA-unwrapping events. Solid lines are Gaussian fits and the fit parameters are summarized in S1 Table. (g-h) Rupture-force distributions of the selected 15–30 nm steps from (g) H3 and (h) CENP-A nucleosome disassembly. Color scheme is the same as in (e). Solid lines are Gaussian fits to the data and the fit parameters are summarized in **S2 Table**.

To study the force-dependent stability of nucleosomes, we measured the unpeeling of the inner-turn DNA from a nucleosome (**Fig 2A**). After assembly of nucleosomes, we linearly increased the applied force on the DNA at a constant slow speed of 0.1 pN/s while the height of the magnetic bead was being monitored (**Fig 2B**). While the force increase resulted in a gradual increase of the bare DNA extension (**Figs 2C and S3A**, black lines), additional discrete jumps in bead height were observed due to the disassembly of individual nucleosomes, which is associated with the release of DNA that was wrapped around the nucleosome (**Figs 2C and S3A**, grey lines with steps highlighted in red). Importantly, no such a stepwise increase was observed for bare DNA molecules (**Figs 2C and S3A**, black lines), showing that the multiple biotin-streptavidin linkage for DNA tethering is strong enough to withstand the applied force. Also, when we incubated DNA with a protein mixture lacking H3/H4 and/or NAP-1, the force-extension curves were identical to that of bare DNA molecules (**S2D Fig**), confirming that the observed stepwise increases were indeed due to the disassembly of nucleosomes and not due to non-specific binding of the proteins to the DNA. Similar stepwise increases were observed for CENP-A nucleosomes (**Figs 2D** and **S3B**).

To quantify the size and the rupture force of the individual disruption events, we next applied a step-finding algorithm (see **S2 Text** for details). The step-size distribution of H3 nucleosome disruption from random DNA reveals a broad population with a majority of steps near 15–30 nm, and a peak at 23 nm with a width (standard deviation) of 4.1 nm (**Fig 2E,** grey bars and **S1 Table**, total N = 622 events). The observed step size is in agreement with previous studies, which have shown disassembly to occur at 25.5 ± 0.4 nm for H3 nucleosomes [51]. Disruption events with larger step-sizes (>30 nm) can be attributed to (i) non-specific DNA sticking to the surface, or (ii) simultaneous disassembly of two or more nucleosome within the time resolution (~100 ms). For the disruption of H3 nucleosomes from centromeric DNA (**S4A Fig**), a similar population in the 15~30 nm range was observed, with a peak at 23 nm with a width of 4.9 nm (**Fig 2E**, red bars, and **S1 Table**, N = 252). From the data, we conclude that the disruption of H3 nucleosomes gives very similar disassembly steps on random and centromeric DNA sequences. An additional population appeared at smaller step sizes (< 15 nm), which dominantly arose from steps in the low-force regime (<20 pN, see **S6 Fig**). At this low-force regime (<20pN), the Brownian fluctuation of the magnetic bead exceeds our spatial resolution (**S7 Fig**), which often results in false-positive detections in the step-finding algorithm (**S6C Fig**). For this reason, these smaller steps were excluded from further analysis.

Next, we analyzed disruption steps of CENP-A nucleosomes (**Figs 2D, S3B and S4B**). As can be seen, CENP-A nucleosomal disassembly was very similar on the random DNA and centromeric DNA, with 24 ± 3.9 nm (mean ± std.) and 22 ± 4.0 nm (mean ± std.) peaks, respectively, in the step-size distributions (**Fig 2F** and **S1 Table,** N = 386 for randDNA; N = 362 for cenDNA). In a direct comparison of the disruption steps for H3 and CENP-A nucleosomes, they are virtually identical with peak values at 24 nm vs. 26 nm for random DNA, and 22nm vs. 22 nm for centromeric DNA. The step-size analysis thus shows that the nucleosome disruption occurs quantitatively similar for H3 and CENP-A nucleosomes, on both random and centromeric DNA.

Next, we analyzed the rupture force, i.e., the precise force at which nucleosome disassembly occurred. We built histograms of the rupture forces from the steps in the range of 15–30 nm (**Fig 2G and 2H**) that comprises the large majority of nucleosome rupture events, while we excluded the false-detection events (10-15nm) and the small fraction of >30nm events that are likely due to double-nucleosome ruptures. We observed disruption events to occur in the force range of 20–40 pN with a peak at 29 ± 5.4 pN (mean ± std.) for H3 nucleosomes on random DNA (**Fig 2G,** grey bars, and **S2 Table**, N = 345). Similarly, H3 disruption from centromeric DNA showed a peak at 28 ± 6.2 pN (mean ± std., **Fig 2G,** red bars, N = 122). The measured rupture forces are slightly higher than the literature values of 10~35 pN [11, 34, 35], because of the lower ionic strength of our measurement buffer in which the electrostatic interaction between the histone proteins and DNA becomes stronger [35]. Indeed, when we increased the ionic strength of our measurement buffer from 50 mM KCl to 200 mM KCl, we observed, as expected, a lower rupture force (23 ± 6.6 pN, **S5 Fig** and **S3 Table**). CENP-A nucleosomes also disassembled in a similar force ranges with a peak at 25 pN ± 4.6 (mean ± std.) for the random DNA and 27 ± 3.2 pN (mean ± std.) from centromeric DNA (**Fig 2H**, N = 204 for randDNA; N = 200 for cenDNA). Overall, we thus were unable to detect significant differences in the rupture force between H3 and CENP-A nucleosomes that were disassembled from either random DNA or centromeric DNA reconstituted chromatin. A secondary population was observed at lower force (~13 pN for CENP-A on both randDNA and cenDNA, and ~15 pN on randDNA and ~8 pN on cenDNA for H3), which can be attributed to (i) outer-turn rupture events, and (ii) false-positive detection in the step-finding algorithm due to the poorer signal-to-noise ratio in the low-force regime in which the Brownian fluctuations of the magnetic beads becomes large (**S6B Fig**). As the main focus of this study is to examine the differences in inner-turn rupture events in higher force-regime–which are clearly distinguishable in the population histograms–we did not include these low-force populations for further analysis.

### Force-clamp measurements shows similar disassembly times for both H3 and CENP-A nucleosomes

**Fig 3:**
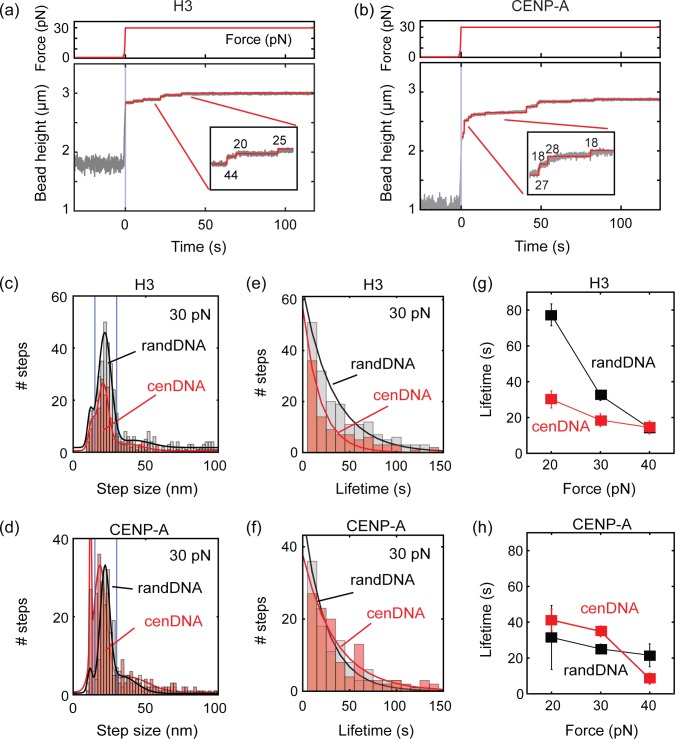
Life time measurements of individual nucleosomes under a constant tension. (a-b) Representative (a) H3 and (b) CENP-A nucleosome disassembly time trace from random DNA sequence under constant force. Red line is a most likely trace deduced by a step-finding algorithm. The determined step sizes (in nm) indicted next to each step. Top panel: the time trace of the force exerted on the DNA. At time 0, the force was increased abruptly from 1 pN to 30 pN. (c-d) Step-size distribution of (c) H3 and (d) CENP-A disassembly from random DNA (grey bars) and centromeric DNA (red bars). Blue lines indicates the 15–30 nm range expected from a single-nucleosome DNA-unwrapping event. Solid lines are multi-Gaussian fits and the fit parameters are summarized in S4 Table (e-f) Life time distributions of the selected 15–30 nm steps for (e) H3 and (f) CENP-A nucleosome disassembly. Color scheme is the same as (c). Solid lines are single-exponential fits to the data and the fit parameters are summarized in **S5 Table**. (g-h) Average life times of (g) H3 and (h) CENP-A nucleosomes obtained from single-exponential fits to the life-time distributions measured at different forces. Error bars are standard errors of the mean.

Another metric of nucleosome stability is the life time residency of nucleosomes (i.e. time spent on the DNA before disassembly) upon applying a constant force. Although the observed rupture forces showed no significant differences, life times before disassembly could be different for H3 and CENP-A nucleosomes if the disassembly process consists of multiple hidden steps [35]. For a further quantitative comparison of the stability of nucleosomes on centromeric DNA and random DNA, we measured the life time of nucleosome disassembly under a constant tension on the DNA. To do so, we first assembled H3 nucleosomes on random DNA as described above and held it at a low force of ~1 pN. Subsequently, we rapidly ramped up the force to 30 pN in a single step at t = 0. We observed an instantaneous increase of the bead height and the DNA extension reached a value close to its B-form contour length of 2.69 μm (**Figs 3A** and **S8A**, grey lines). While the applied force was kept constant at 30 pN, we observed small stepwise increases of the bead height in time. Using the step-finding algorithm, we identified the size and the time delay of individual steps (**Figs 3A** and **S8A**, red line). Similar stepwise increases in the bead height were observed and analyzed for CENP-A nucleosomes (**Figs 3B** and **S8B**) and also for the centromeric DNA (**S9 Fig**).

Consistent to the rupture-force experiments described above, we observed a step-size distribution for H3 nucleosomes with a peak at 23 ± 2.5 nm and 20 ± 3.0, from random DNA and centromeric DNA, respectively (**Fig 3C** and **S3 Table**). CENP-A nucleosomes also consistently showed peaks at 21 ± 3.5 nm and 21 ± 2.7 nm for the random DNA and centromeric DNA, respectively (**Fig 3D**). To determine the stability of the nucleosome under tension, we collected all steps of 15–30 nm size and assembled histograms of the life-time distributions. These showed a single-exponential distribution as expected for a single-step disassembly process (**Fig 3E and 3F**). We repeated these measurements for different applied forces at 20 pN and 40pN (**S10 Fig**), and obtained the same single exponential distributions of life times from the steps of 15–30 nm (**S11** and **S12 Figs**). The mean life times determined from these fits are summarized in **Fig 3G and 3H**. Interestingly, H3 nucleosomes tend to stay slightly longer on random DNA than on centromeric DNA; conversely, CENP-A nucleosomes display a marginally increased stability on centromeric DNA. For example, at 30 pN, H3 nucleosomes disassembled with a mean life time of, τ = 33 ± 3 sec (mean ± s.e.) from random DNA. This is ~1.7 times longer than that of centromeric DNA, τ = 19 ± 4 sec (mean ± s.e.). In contrast, CENP-A nucleosomes tend to stay longer on centromeric DNA, τ = 35 ± 3 sec (mean ± s.e.), than on random DNA, τ = 25 ± 3 sec (mean ± s.e.). These data suggest that CENP-A nucleosomes might have a slight preference for retention on centromeric DNA over random DNA sequences.

## Discussion

In this report, we compared the stability of CENP-A and H3 nucleosomes on random-sequence DNA and centromeric alpha-satellite-containing DNA, in order to test the hypothesis that CENP-A nucleosomes on their cognate centromeric DNA may have special properties when challenged with force-induced disruption.

Contrary to the hypothesis of enhanced stability of CENP-A nucleosomes, our magnetic tweezers data show that the force response of H3 and CENP-A nucleosomes are very similar, and the results are generally independent on the type of DNA used, i.e. random versus centromeric DNA. In almost all the metrics used to quantify nucleosome stability in these assays, we find very similar results for H3 and CENP-A nucleosomes. Although the observed similarity of CENP-A and H3 nucleosomes is surprising, it is supported by their near-identical octameric crystallographic structures [19, 52], and is consistent with the alternative idea that CENP-A serves predominantly as an epigenetic mark, a binding platform for other kinetochore proteins to form an intricate 3-dimensional array of intertwined fibers, which is, in turn more likely to provide the appropriate balance between resilience and flexibility needed to undergo mitosis [53–57].

Our data shows that the intrinsic stability of CENP-A nucleosomes is no better or worse than the stability of canonical H3 nucleosomes. These data generally support the hypothesis, proposed nearly a decade ago, that CENP-A nucleosomes might be intrinsically pliable and easy to disassemble [58]. Because CENP-A can recruit a myriad of kinetochore proteins, however, it might be evolutionarily beneficial to promote their disassembly at ectopic sequences in the absence of kinetochore proteins, thereby preventing fortuitous seeding of new centromeric domains in the absence of correct kinetochore partners. This view is further favored by the observation that the lifetimes reveal that CENP-A has a minor advantage over H3 on centromeric DNA: while H3 is slightly more stable on non-centromeric DNA, the reverse is true for CENP-A (**Fig 3G and 3H**). These observations are also consistent with previous reports showing that the vast majority of natural human centromeres are composed of alpha-satellite DNA sequences [59, 60], and with the recent finding that active centromeres on human artificial chromosomes require alpha-satellite repeats [61, 62]. Thus, it is tempting to speculate that *in vivo*, CENP-A may have a very slight advantage over H3 during chaperone-mediated assembly on centromeric DNA sequences [63, 64], which may potentially contribute kinetically to rapid deposition of CENP-A arrays from which H3 is competitively and temporally excluded during early G1 phase [65, 66].

We cautiously note that these experiments have been carried out on individual nucleosomes, and we do not exclude the possibility of a constructive synergetic effect when CENP-A nucleosomes forms an array of nucleosomes guided by the alpha-satellite repeats [53, 54], interspersed with CENP-B and CENP-C. Indeed, in the case of the human centromere, multiple CENP-A nucleosomes establish connections to kinetochore proteins, which might amplify the very slight differences seen here between individual CENP-A vs. H3 nucleosomes. Lacking direct experimental evidence on non-canonical conformations of CENP-A (in the absence of presence of key kinetochore partners), we cannot conclude whether such non-octameric species might or might not have a different response to force. Together, these data have implications for the function of CENP-A *in vivo*, demonstrating that its primary role is to serve as a network node, organizing kinetochore proteins into a three dimensional matrix which in turn provides resilience to the centromere [54, 56, 67]. Thus, while this report is focused on characterizing the stability of individual mono-nucleosomes, force resistance of fully packed centromeric chromatin fibers, bound to the key trifecta of inner kinetochore partners CENP-C, CENP-B and CENP-N [68–71], presents an interesting extension for future research.

In conclusion, the single-molecule experiments presented here demonstrate that human H3 and CENP-A nucleosomes have very similar force-dependent stabilities and, secondly, that human centromeric DNA consisting of alpha-satellite repeats does not confer special structural properties on individual CENP-A nucleosomes *in vitro*. These data provide insights into the mechanistic basis for which features of CENP-A nucleosomes might be critical for the structural foundation of centromere *in vivo*.

## Supporting Information

S1 Fig A schematic diagram of the magnetic tweezers. The magnetic tweezers setup consists of the three parts: 1) a flow cell with microfluidic control of the samples, 2) a pair of magnets on motorized stages for force and rotation control of the tethered DNA, and 3) an imaging system with an objective lens and a CCD camera for magnetic bead tracking. In the flow cell, DNA molecules are covalently bound on a glass slide surface via DBCO-Azide click chemistry. The other end of the DNA is bound to a magnetic bead via a biotin-streptavidin linker. The magnetic bead is then pulled by a pair of magnet placed above the flow cell. Two holes on the flow cell serves as inlet and outlet for buffer exchange. A red LED illuminates the magnetic beads which are then imaged by the objective lens and CCD camera.
(PDF)

S2 Fig Time traces and force-extension curves in the absence of H3/CENP-A and H4. (a-b) DNA-extension time trace in the presence of (a) H2A and H2B but not H3/H4 and NAP-1 and (b) H2A and H2B and NAP-1, but not H3/H4. The protein concentrations were the same as in Fig 1B. The DNA extension did not change after flowing in the proteins, showing that no nucleosome assembly occurs. Thin line shows raw data at 50 Hz bandwidth; thick blue line is the moving average with 1 s time window. (c) MNase digestion assay. Each sample represents a different histone/NAP-1 combination that was digested with MNase for either 0, 3, and 10 min as indicated in the figure. When both histones and NAP-1 are present, we observe a clear band at ~150 bp (~120bp) for H3 (CENP-A). Note also that we do not observe any clear bands below ~100 bp which would arise from other conformations such as tetrasomes. (d) Force-extension curves for H2A and H2B (green), H2A, H2B and NAP-1 (red), and bare DNA (blue). The curves are identical and no stepwise increase in the DNA extension observed.
(PDF)

S3 Fig Force-extension curves measured with random DNA. (a-b) Examples of force-extension curve measured before (black line) and after (grey line) assembly of (a) H3 and (b) CENP-A nucleosomes on the random DNA. Steps identified by the step-finding algorithm are highlighted in red with the step sizes revealed in nm.
(PDF)

S4 Fig Force-extension curves measured with centromeric DNA. (a-b) Examples of force-extension curve measured before (black line) and after (grey line) assembly of (a) H3 and (b) CENP-A nucleosomes on cenDNA. Steps identified by the step-finding algorithm are highlighted in red with the step sizes revealed in nm.
(PDF)

S5 Fig Force-ramp measurements at high salt condition. (a) Step-size and (b) Rupture force distribution of H3 nucleosome disruption events measured at 200 mM KCl. Solid lines are multi-Gaussian fits to the data set and the fit parameters are summarized in S3 Table. (c) Rupture forces in (b) plotted against their step-size in (a).
(PDF)

S6 Fig Step size versus rupture force. (a-b) Rupture forces of (a) H3 and (b) CENP-A nucleosomes identified from the step-finding algorithm, plotted against their corresponding step sizes. Black circles: Random DNA, Red circles: cenDNA. (c) False-positive detection from the bare DNA molecules (random DNA). Unlike the cases in (a) and (b), the data for the bare DNA molecule do not show any noticeable population in the range of 15–30 nm steps at a force range of 20–40 pN.
(PDF)

S7 Fig Force-dependent noise level in bead height measurement. (a-d) Bead heights plotted against force from bare DNA tethers (upper panels, black lines). Standard deviations of the bead heights calculated at each force are plotted in bottom panels (red lines).
(PDF)

S8 Fig Time traces of the bead height under a constant force measured on random DNA. (a-b) Example of (a) H3 and (b) CENP-A nucleosome disassembly traces from the random DNA. At t = 0, the force was increased suddenly from 1pN to 30pN. The DNA extension instantaneously responded to the pulling force. Afterwards, small stepwise increases (<100nm) of the extension were observed. The most likely trajectory found from the step-finding algorithm is plotted in red. Identified step sizes are indicated in nm. (Top) The force trace shows the stepwise change in the force.
(PDF)

S9 Fig Time traces of the bead height under a constant force measured on centromeric DNA. (a-b) Examples of (a) H3 and (b) CENP-A nucleosome disassembly traces from centromeric DNA. At t = 0, the force was increased suddenly from 1pN to 30pN. The DNA extension instantaneously responded to the pulling force. Afterwards, small stepwise increases (<100nm) of the extension were observed. The most likely trajectory found from the step-finding algorithm is plotted in red. Identified step sizes are indicated in nm. (Top) The force trace shows the stepwise change in the force.
(PDF)

S10 Fig Step size distributions from the constant force-pulling measurements. (a-c) Step size distributions of H3 nucleosome disruptions from random DNA (top panels) and centromeric DNA (bottom panels) measured at a constant force of (a) 20pN, (b) 30 pN and (c) 40 pN. Solid lines are multi-Gaussian fits and the fit parameters are summarized in S4 Table. (d-f) Step size distributions of the CENP-A nucleosome disruptions from random DNA (top panels) and centromeric DNA (bottom panels), measured at a constant force of (d) 20 pN, (e) 30 pN, and (f) 40 pN. Solid lines are multi-Gaussian fits and the fit parameters are summarized in S4 Table.
(PDF)

S11 Fig Scattered plots of step size vs. life time from the constant-force pulling measurements. (a-c) Life times of H3 nucleosome disruptions from the constant force pulling measurements at (a) 20 pN, (b) 30 pN and (c) 40 pN, plotted against the corresponding step sizes. Black circles: Random DNA, Red circles: cenDNA. (d-f) Life times of CENP-A nucleosome disruptions against step size, measured at a constant force of (d) 20 pN, (e) 30 pN, and (f) 40 pN.
(PDF)

S12 Fig Life-time distributions from the constant force-pulling measurements. (a-c) Life-time distributions of H3 nucleosome disruptions from random DNA (grey bars) and cenDNA (red bars) measured at a constant force of (a) 20pN, (b) 30 pN and (c) 40 pN. Solid lines are single-exponential fits to the data sets and the fit parameters are summarized in S5 Table. (d-f) Life-time distributions of the CENP-A nucleosome disruptions, measured at a constant force of (d) 20 pN, (e) 30 pN, and (f) 40 pN. Solid lines are single-exponential fits to the data sets and the fit parameters are summarized in S5 Table.
(PDF)

S1 Table Multi-Gaussian fit results of step size distribution from force-ramp data in Fig 2E and 2F. (PDF)

S2 Table Multi-Gaussian fit results of rupture force distribution from force-ramp data in Fig 2G and 2H. (PDF)

S3 Table Multi-Gaussian fit results of rupture force distribution from force-ramp data in S5 Fig. (PDF)

S4 Table Multi-Gaussian fit results of step size distribution from force-ramp data in Fig 3C and 3D and S10 Fig. (PDF)

S5 Table Single-exponential fit results of life-time distribution from force-clamp data in Fig 3E–3G and S12 Fig. (PDF)

S1 Text DNA sequences. (PDF)

S2 Text Step-finding algorithm. (PDF)
