# Supplementary figures and images for: CENP-A and H3 Nucleosomes Display a Similar Stability to Force-Mediated Disassembly
Source: PLoS One. 2016 Nov 7;11(11):e0165078. doi: 10.1371/journal.pone.0165078 (PMC5098787; doi:10.1371/journal.pone.0165078)

S1 Fig.

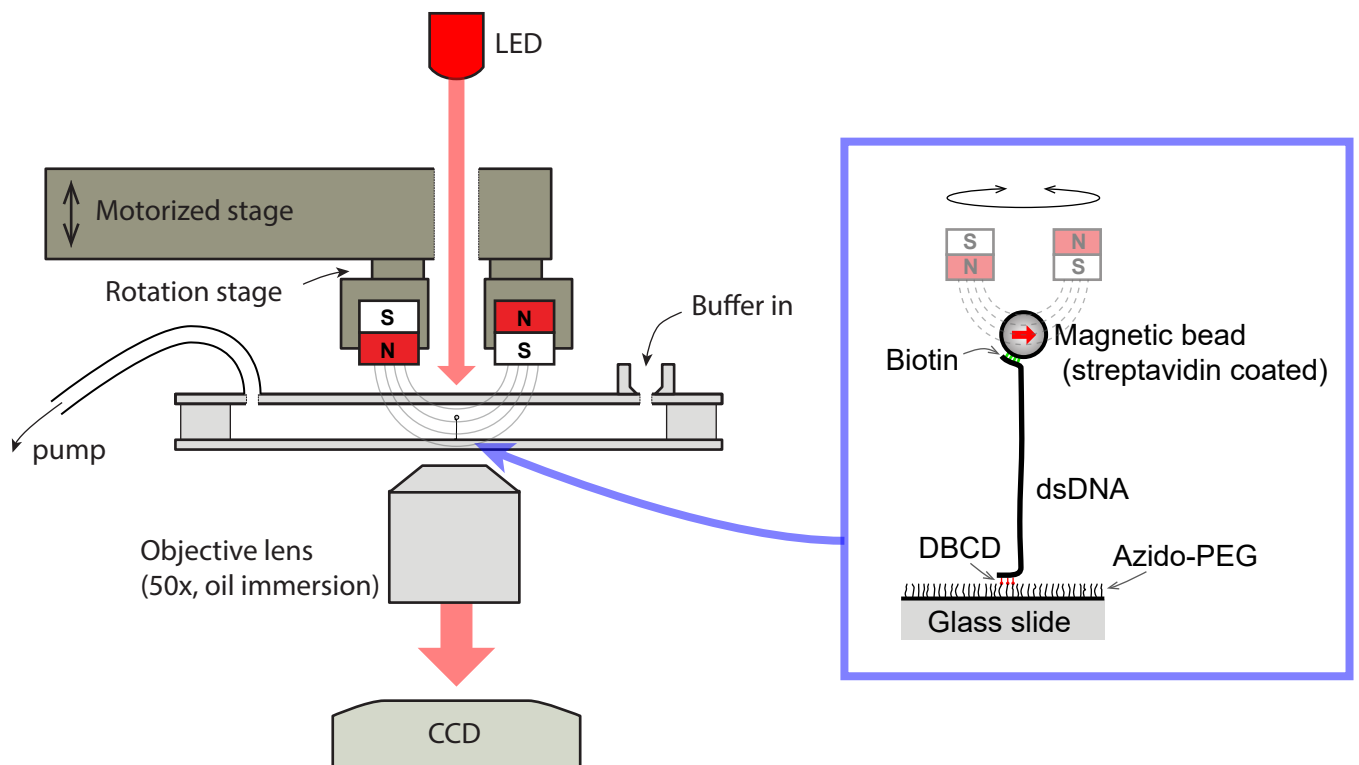

Supplement: S1 Fig — The magnetic tweezers setup consists of the three parts: 1) a flow cell with microfluidic control of the samples, 2) a pair of magnets on motorized stages for force and rotation control of the tethered DNA, and 3) an imaging system with an objective lens and a CCD camera for magnetic bead tracking. In the flow cell, DNA molecules are covalently bound on a glass slide surface via DBCO-Azide click chemistry. The other end of the DNA is bound to a magnetic bead via a biotin-streptavidin linker. The magnetic bead is then pulled by a pair of magnet placed above the flow cell. Two holes on the flow cell serves as inlet and outlet for buffer exchange. A red LED illuminates the magnetic beads which are then imaged by the objective lens and CCD camera. (PDF) [file pone.0165078.s001.pdf]

S2 Fig.

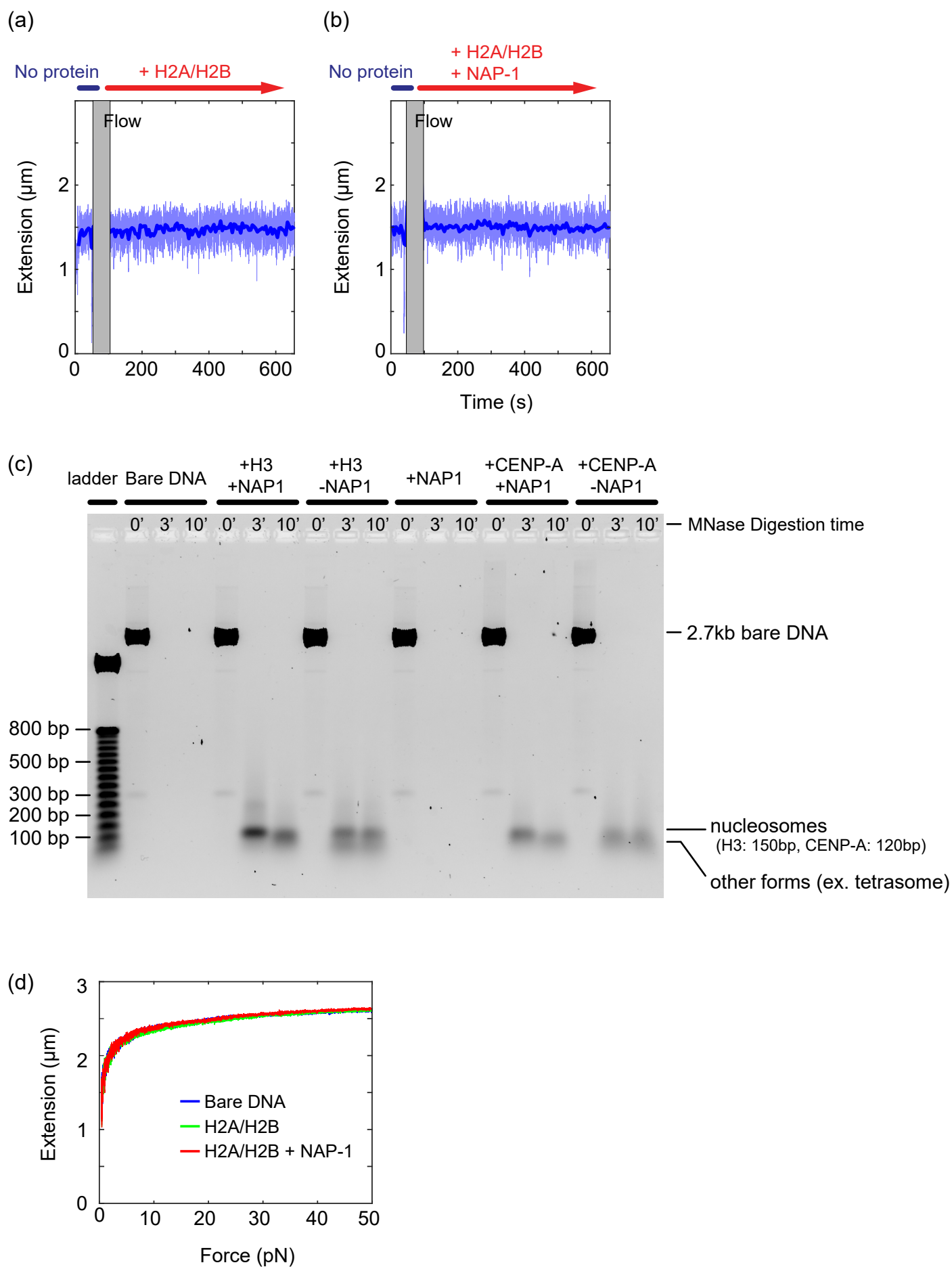

Supplement: S2 Fig — (a-b) DNA-extension time trace in the presence of (a) H2A and H2B but not H3/H4 and NAP-1 and (b) H2A and H2B and NAP-1, but not H3/H4. The protein concentrations were the same as in Fig 1B. The DNA extension did not change after flowing in the proteins, showing that no nucleosome assembly occurs. Thin line shows raw data at 50 Hz bandwidth; thick blue line is the moving average with 1 s time window. (c) MNase digestion assay. Each sample represents a different histone/NAP-1 combination that was digested with MNase for either 0, 3, and 10 min as indicated in the figure. When both histones and NAP-1 are present, we observe a clear band at ~150 bp (~120bp) for H3 (CENP-A). Note also that we do not observe any clear bands below ~100 bp which would arise from other conformations such as tetrasomes. (d) Force-extension curves for H2A and H2B (green), H2A, H2B and NAP-1 (red), and bare DNA (blue). The curves are identical and no stepwise increase in the DNA extension observed. (PDF) [file pone.0165078.s002.pdf]

S4 Fig.

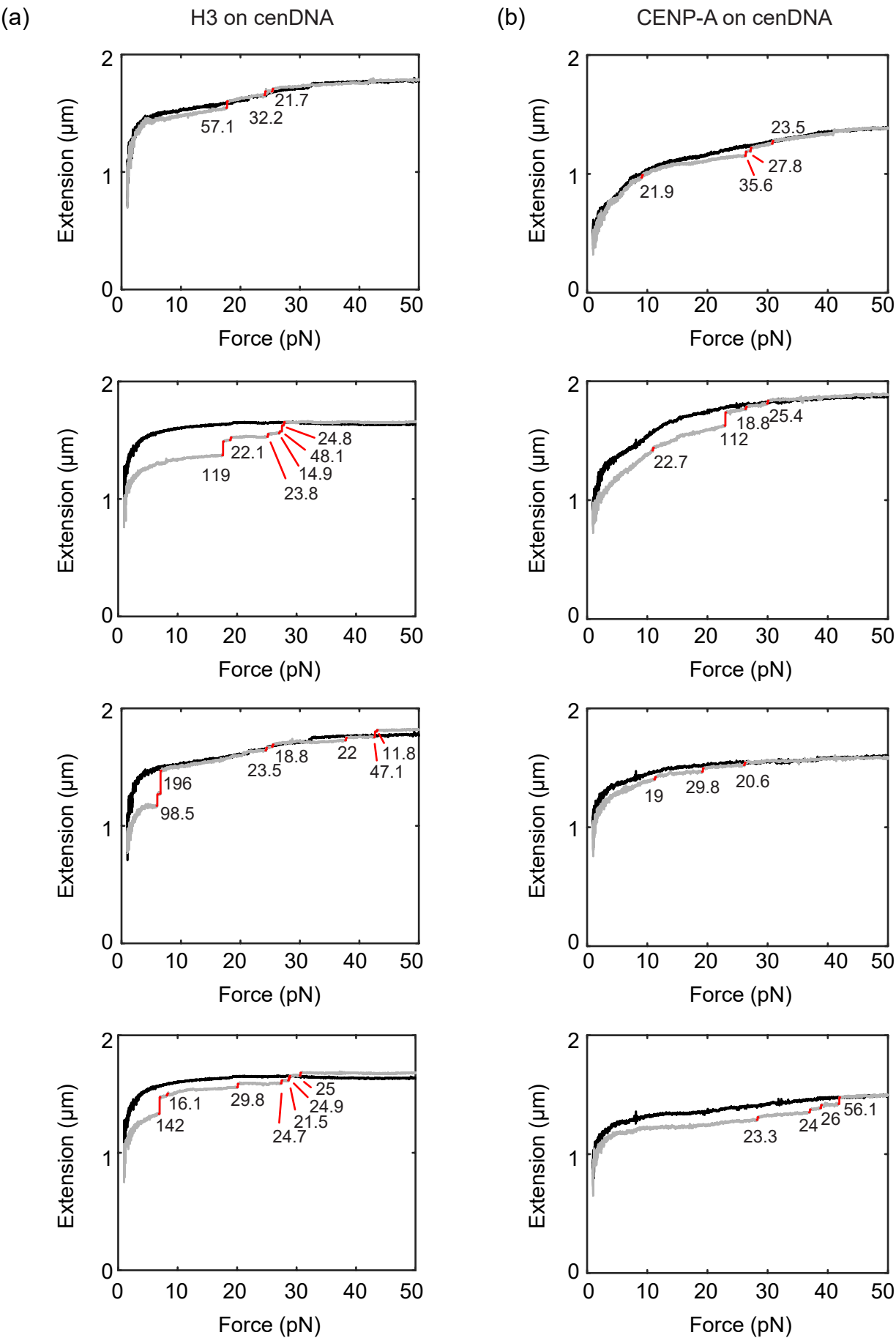

Supplement: S4 Fig — (a-b) Examples of force-extension curve measured before (black line) and after (grey line) assembly of (a) H3 and (b) CENP-A nucleosomes on cenDNA. Steps identified by the step-finding algorithm are highlighted in red with the step sizes revealed in nm. (PDF) [file pone.0165078.s004.pdf]

S5 Fig.

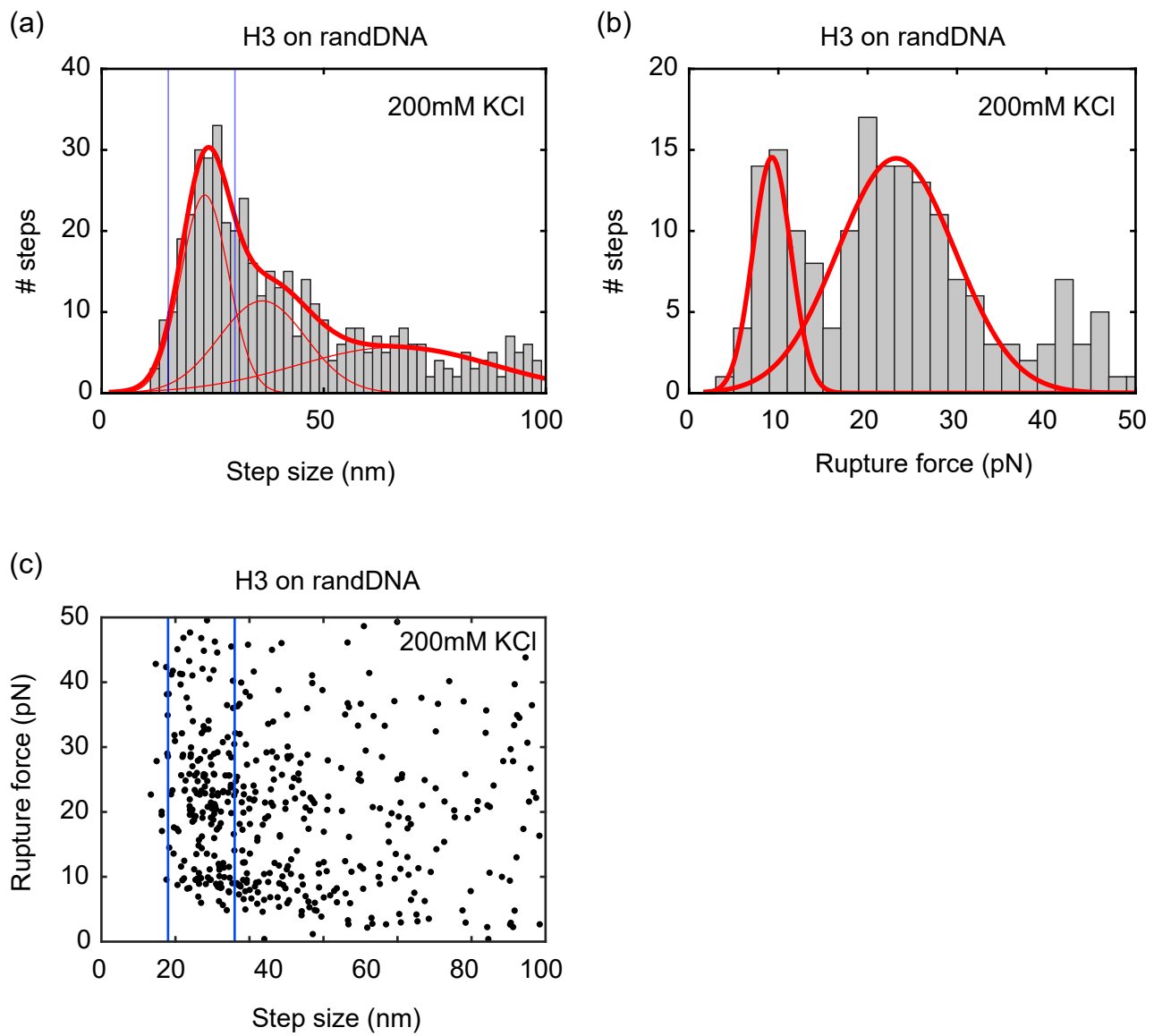

Supplement: S5 Fig — (a) Step-size and (b) Rupture force distribution of H3 nucleosome disruption events measured at 200 mM KCl. Solid lines are multi-Gaussian fits to the data set and the fit parameters are summarized in S3 Table. (c) Rupture forces in (b) plotted against their step-size in (a). (PDF) [file pone.0165078.s005.pdf]

S6 Fig.

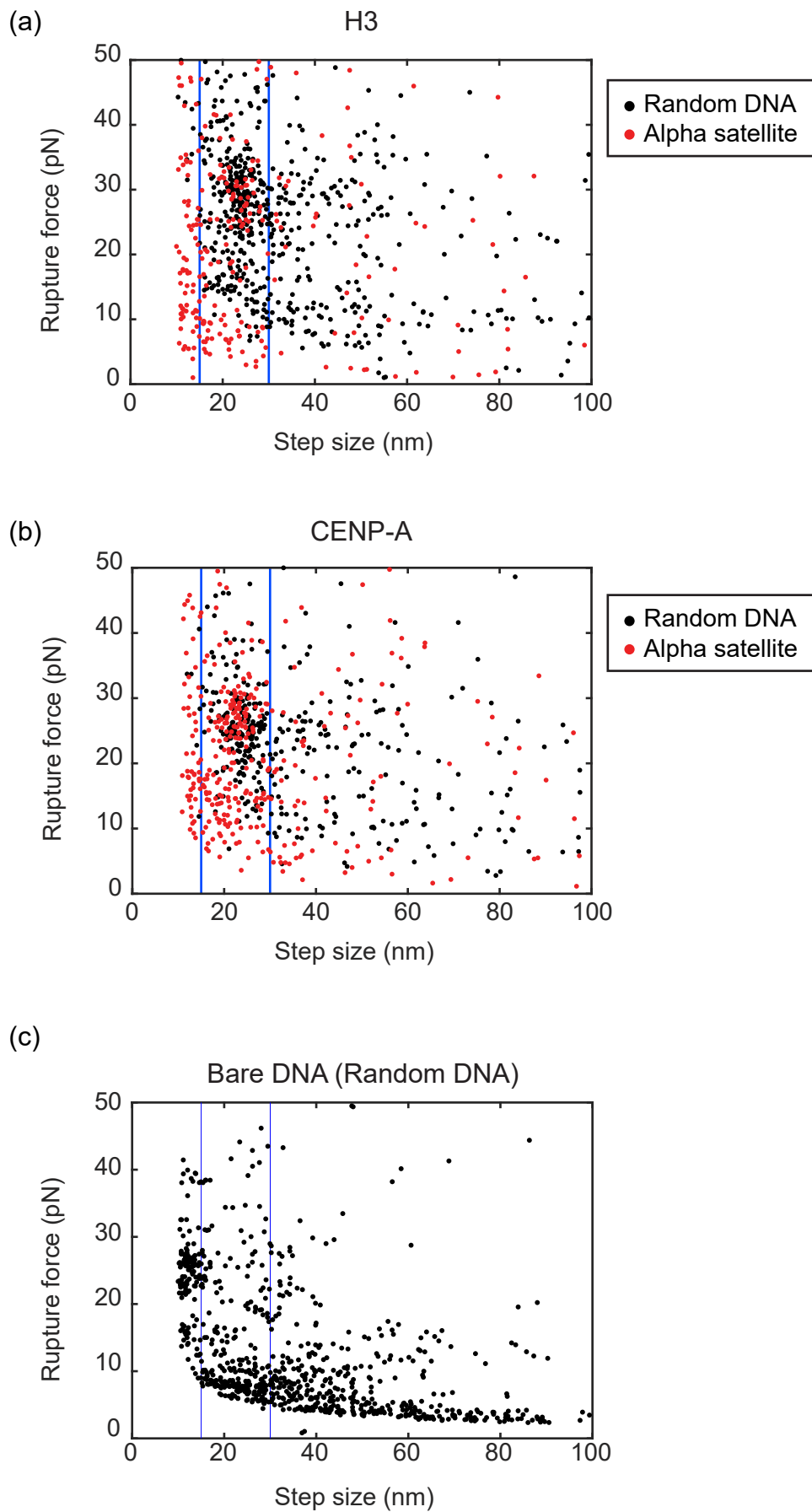

Supplement: S6 Fig — (a-b) Rupture forces of (a) H3 and (b) CENP-A nucleosomes identified from the step-finding algorithm, plotted against their corresponding step sizes. Black circles: Random DNA, Red circles: cenDNA. (c) False-positive detection from the bare DNA molecules (random DNA). Unlike the cases in (a) and (b), the data for the bare DNA molecule do not show any noticeable population in the range of 15–30 nm steps at a force range of 20–40 pN. (PDF) [file pone.0165078.s006.pdf]

S7 Fig.

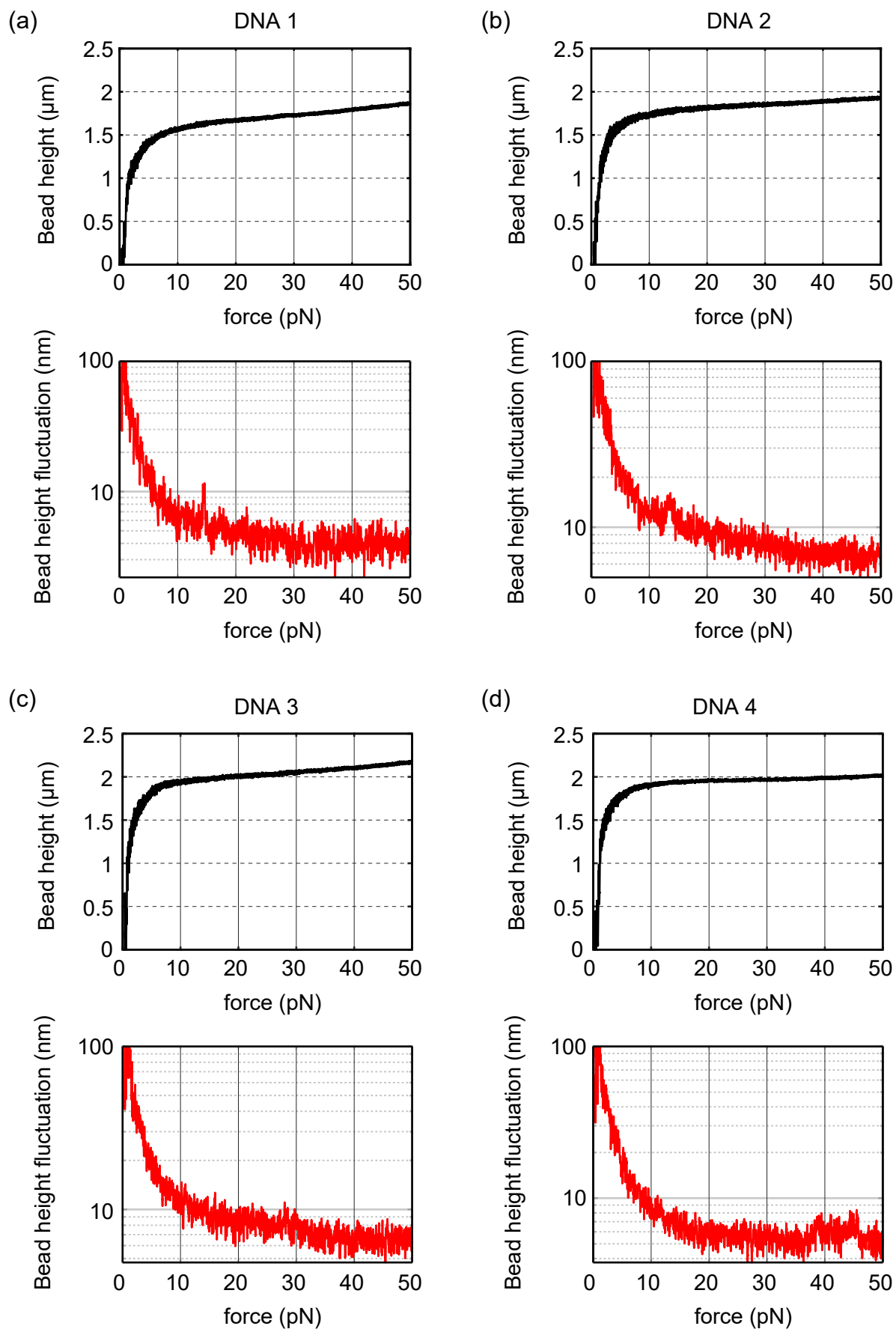

Supplement: S7 Fig — (a-d) Bead heights plotted against force from bare DNA tethers (upper panels, black lines). Standard deviations of the bead heights calculated at each force are plotted in bottom panels (red lines). (PDF) [file pone.0165078.s007.pdf]

S8 Fig.

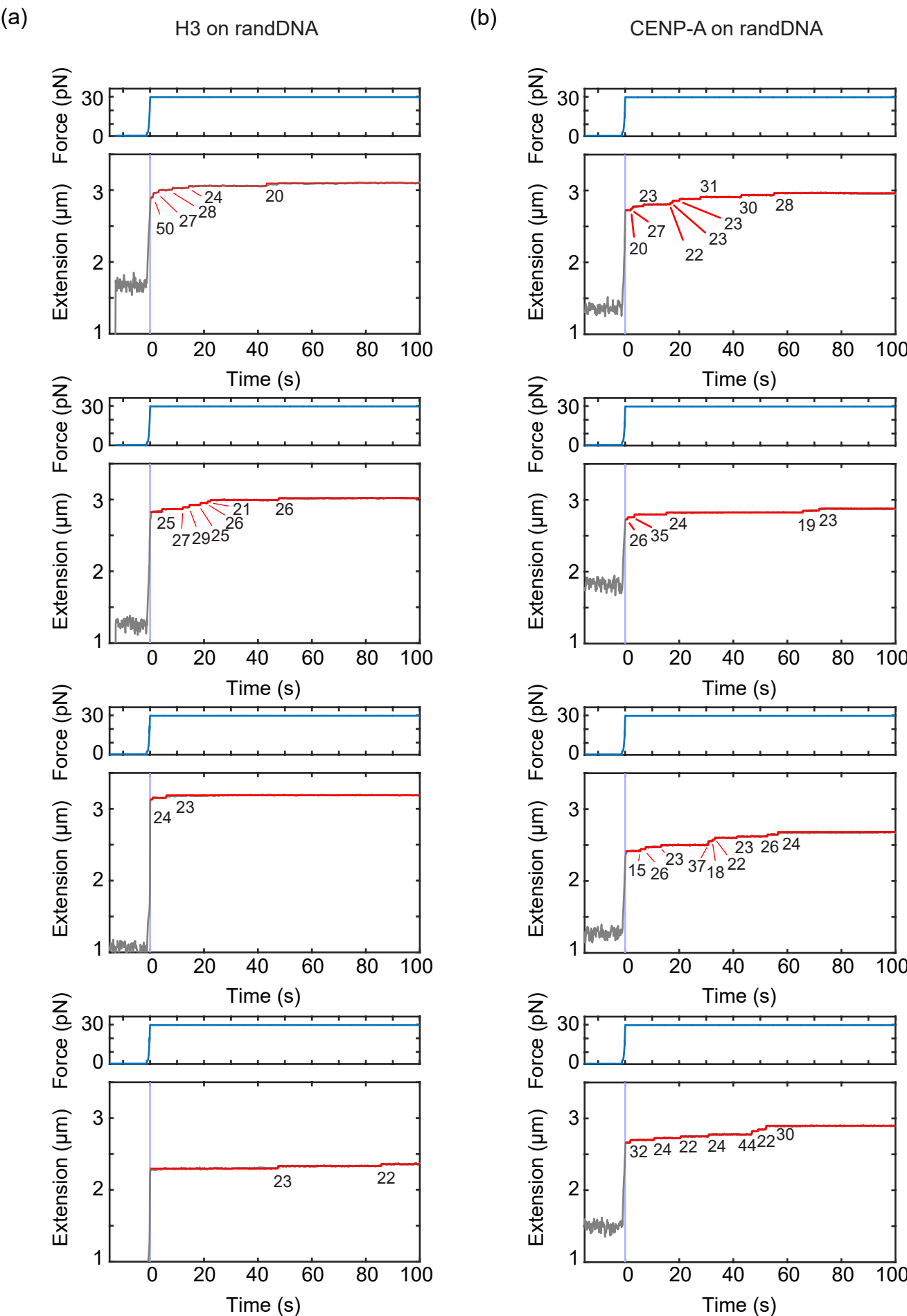

Supplement: S8 Fig — (a-b) Example of (a) H3 and (b) CENP-A nucleosome disassembly traces from the random DNA. At t = 0, the force was increased suddenly from 1pN to 30pN. The DNA extension instantaneously responded to the pulling force. Afterwards, small stepwise increases (<100nm) of the extension were observed. The most likely trajectory found from the step-finding algorithm is plotted in red. Identified step sizes are indicated in nm. (Top) The force trace shows the stepwise change in the force. (PDF) [file pone.0165078.s008.pdf]

S9 Fig.

(a)

H3 on cenDNA

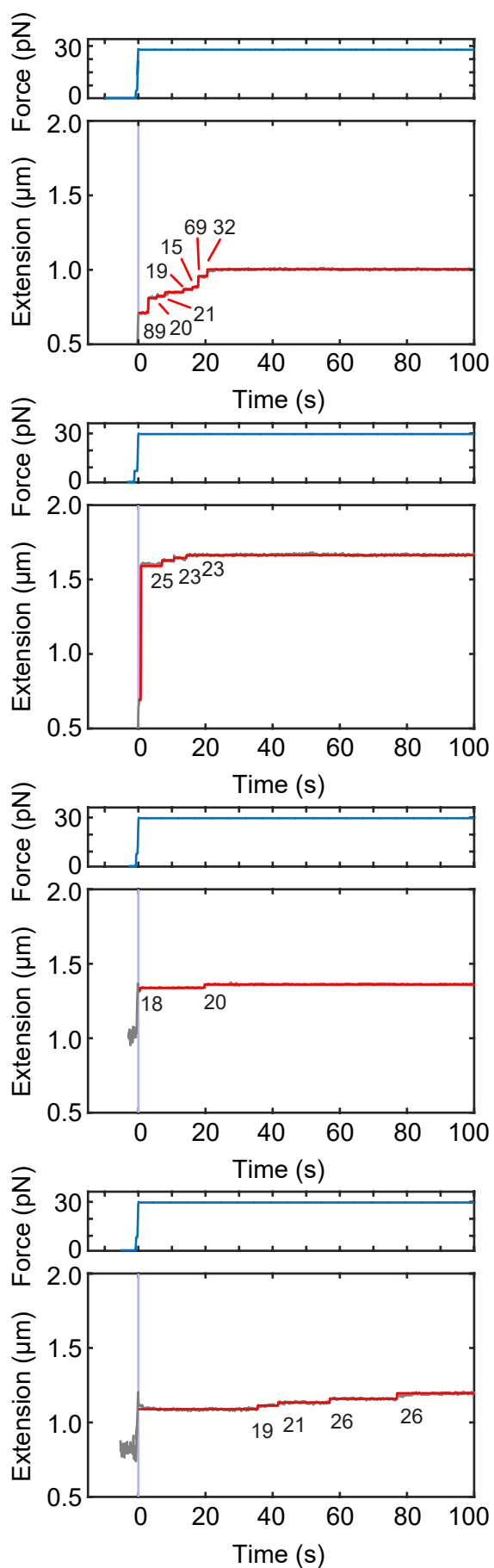

(b)

## CENP-A on cenDNA

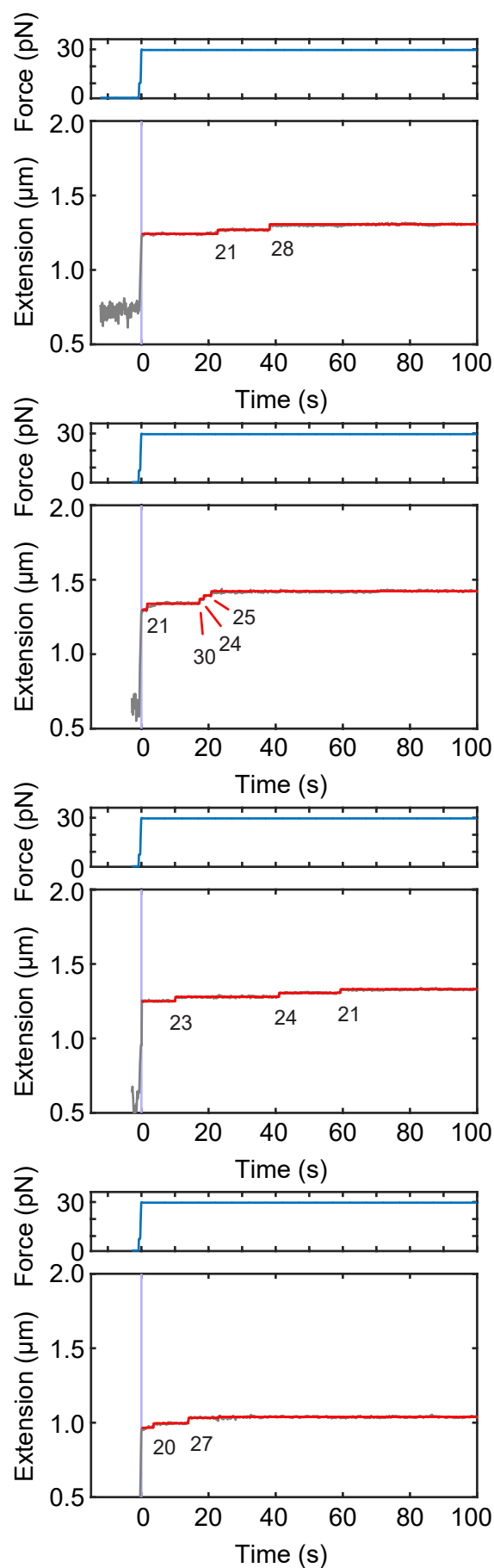

Supplement: S9 Fig — (a-b) Examples of (a) H3 and (b) CENP-A nucleosome disassembly traces from centromeric DNA. At t = 0, the force was increased suddenly from 1pN to 30pN. The DNA extension instantaneously responded to the pulling force. Afterwards, small stepwise increases (<100nm) of the extension were observed. The most likely trajectory found from the step-finding algorithm is plotted in red. Identified step sizes are indicated in nm. (Top) The force trace shows the stepwise change in the force. (PDF) [file pone.0165078.s009.pdf]

S10 Fig.

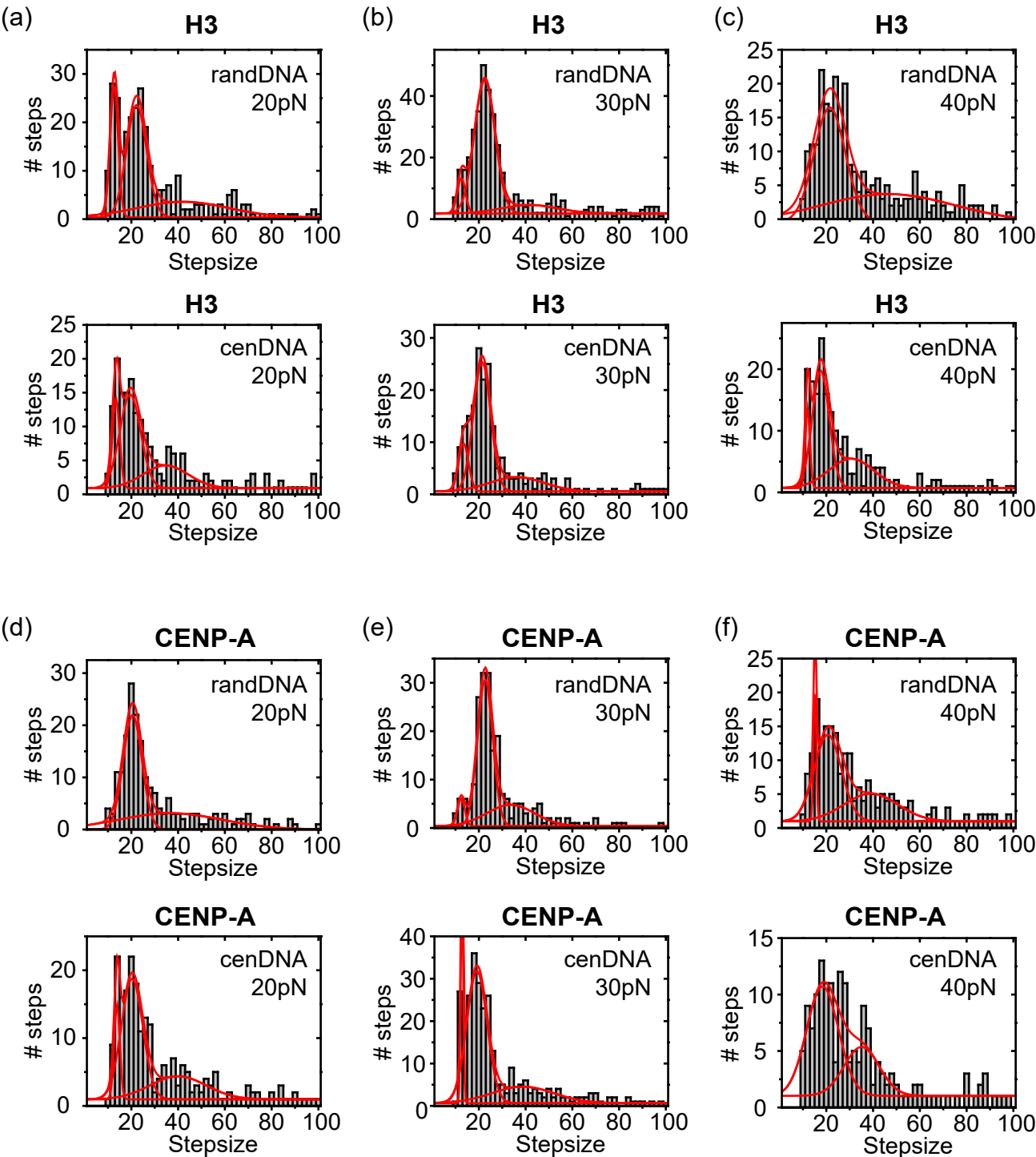

Supplement: S10 Fig — (a-c) Step size distributions of H3 nucleosome disruptions from random DNA (top panels) and centromeric DNA (bottom panels) measured at a constant force of (a) 20pN, (b) 30 pN and (c) 40 pN. Solid lines are multi-Gaussian fits and the fit parameters are summarized in S4 Table. (d-f) Step size distributions of the CENP-A nucleosome disruptions from random DNA (top panels) and centromeric DNA (bottom panels), measured at a constant force of (d) 20 pN, (e) 30 pN, and (f) 40 pN. Solid lines are multi-Gaussian fits and the fit parameters are summarized in S4 Table. (PDF) [file pone.0165078.s010.pdf]

S11 Fig.

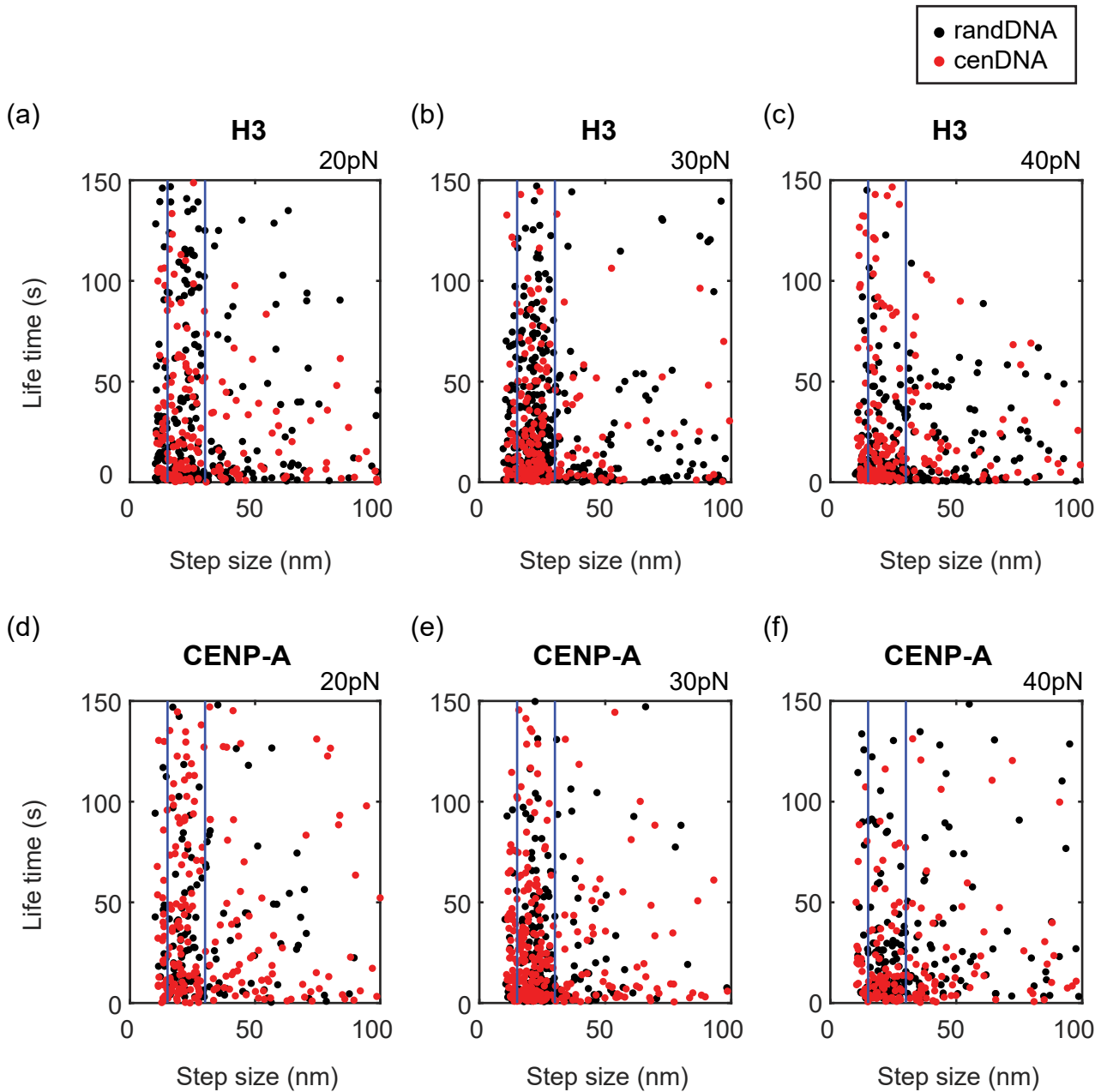

Supplement: S11 Fig — (a-c) Life times of H3 nucleosome disruptions from the constant force pulling measurements at (a) 20 pN, (b) 30 pN and (c) 40 pN, plotted against the corresponding step sizes. Black circles: Random DNA, Red circles: cenDNA. (d-f) Life times of CENP-A nucleosome disruptions against step size, measured at a constant force of (d) 20 pN, (e) 30 pN, and (f) 40 pN. (PDF) [file pone.0165078.s011.pdf]

S12 Fig.

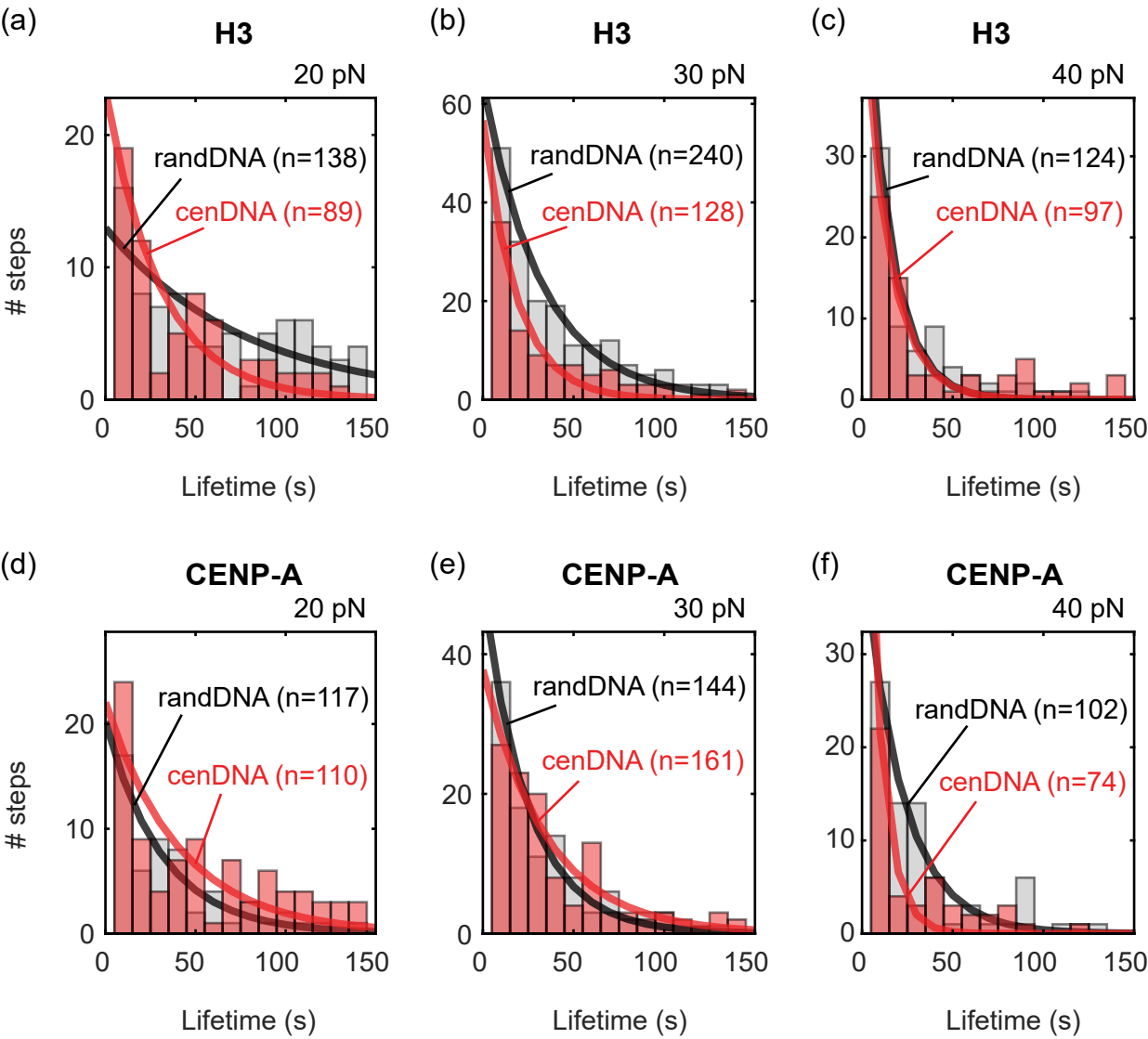

Supplement: S12 Fig — (a-c) Life-time distributions of H3 nucleosome disruptions from random DNA (grey bars) and cenDNA (red bars) measured at a constant force of (a) 20pN, (b) 30 pN and (c) 40 pN. Solid lines are single-exponential fits to the data sets and the fit parameters are summarized in S5 Table. (d-f) Life-time distributions of the CENP-A nucleosome disruptions, measured at a constant force of (d) 20 pN, (e) 30 pN, and (f) 40 pN. Solid lines are single-exponential fits to the data sets and the fit parameters are summarized in S5 Table. (PDF) [file pone.0165078.s012.pdf]
