# Supplementary material for: CENP-A and H3 Nucleosomes Display a Similar Stability to Force-Mediated Disassembly
Source: PLoS One. 2016 Nov 7;11(11):e0165078. doi: 10.1371/journal.pone.0165078 (PMC5098787; doi:10.1371/journal.pone.0165078)
Supplement: S3 Fig — (a-b) Examples of force-extension curve measured before (black line) and after (grey line) assembly of (a) H3 and (b) CENP-A nucleosomes on the random DNA. Steps identified by the step-finding algorithm are highlighted in red with the step sizes revealed in nm. (PDF) [file pone.0165078.s003.pdf]

S3 Fig.

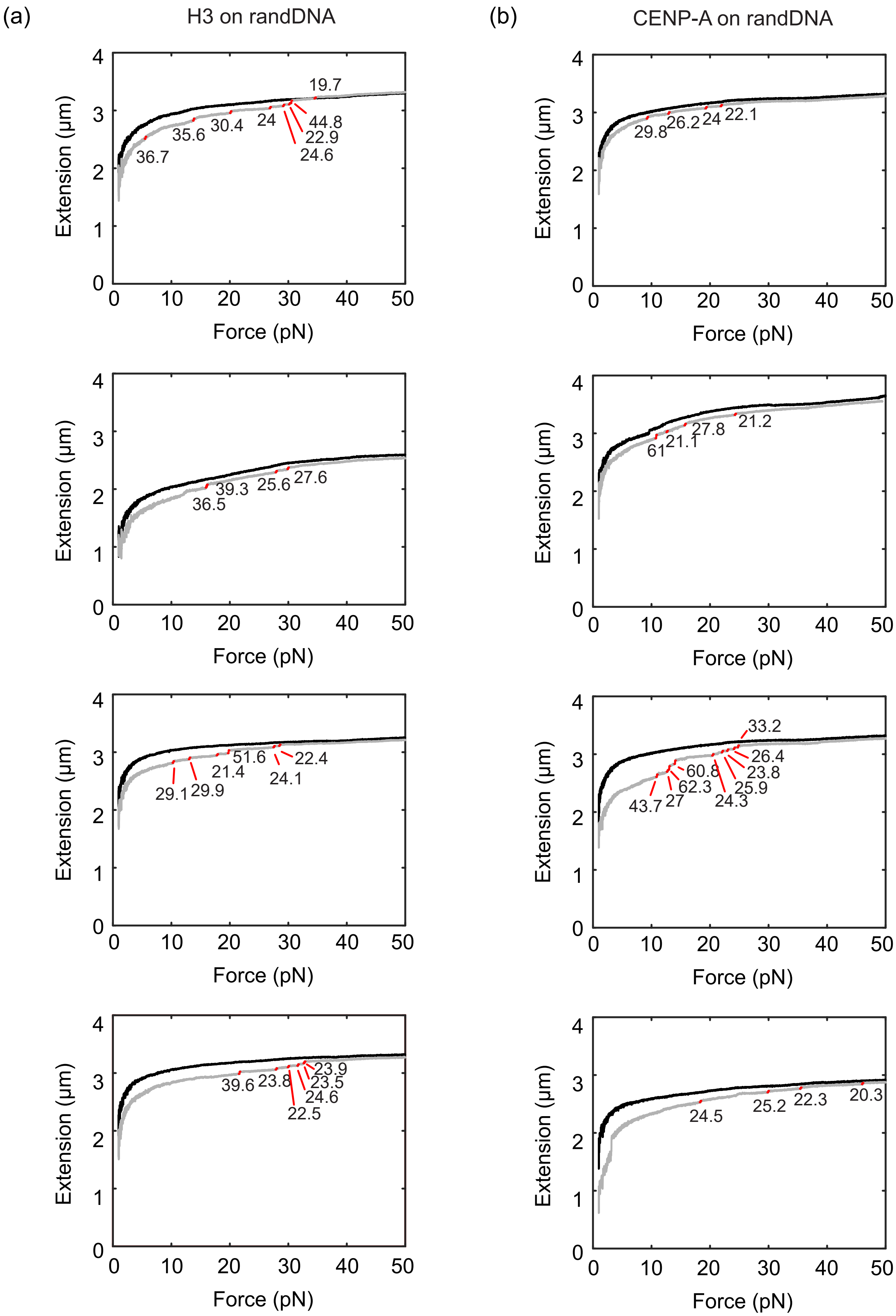

(a)
H3 on randDNA
(b)
CENP-A on randDNA
Extension (μm)
Force (pN)
19.7
44.8
22.9
24.6
36.7
35.6
30.4
24
29.8
26.2
24
22.1
39.3
25.6
27.6
36.5
61
21.1
27.8
21.2
51.6
22.4
21.4
24.1
29.1
29.9
33.2
26.4
60.8
23.8
62.3
25.9
43.7
27
24.3
39.6
23.8
23.9
23.5
24.6
22.5
24.5
25.2
22.3
20.3
