# Supplementary material for: CENP-A and H3 Nucleosomes Display a Similar Stability to Force-Mediated Disassembly
Source: PLoS One. 2016 Nov 7;11(11):e0165078. doi: 10.1371/journal.pone.0165078 (PMC5098787; doi:10.1371/journal.pone.0165078)
Supplement: S1 Table — (PDF) [file pone.0165078.s013.pdf]

SUPPLEMENTARY TABLE 1

Multi-Gaussian fit parameters of step size distribution from force-ramp data in Fig. 2e and 2f

Protein	DNA	Peak1 (nm)	Peak2 (nm)	Peak3 (nm)
H3*	Random		23±4.1	38±15
H3	Centromeric	13±2.1	23±4.9	52±20
CA*	Random		24±3.9	42±19
CA	Centromeric	13±1.6	22±4.0	34±13

* Three-Gaussian peak fitting does not reduce chi-square value comparing to two-Gaussian peak fitting.

** Error bars are standard deviation of the population distribution.
