# Supplementary material for: CENP-A and H3 Nucleosomes Display a Similar Stability to Force-Mediated Disassembly
Source: PLoS One. 2016 Nov 7;11(11):e0165078. doi: 10.1371/journal.pone.0165078 (PMC5098787; doi:10.1371/journal.pone.0165078)
Supplement: S2 Table — (PDF) [file pone.0165078.s014.pdf]

SUPPLEMENTARY TABLE 2

Multi-Gaussian fit parameters of rupture force distribution from force-ramp data in Fig. 2g and 2h

Protein	DNA	Peak1 (pN)	Peak2 (pN)
H3	Random	15±2.3	29±5.4
H3	Centromeric	7.8±2.8	28±6.2
CA	Random	13±0.5	25±4.6
CA	Centromeric	13±4.4	27±3.2

* Error bars are standard deviation of the population distribution.
