# Supplementary material for: CENP-A and H3 Nucleosomes Display a Similar Stability to Force-Mediated Disassembly
Source: PLoS One. 2016 Nov 7;11(11):e0165078. doi: 10.1371/journal.pone.0165078 (PMC5098787; doi:10.1371/journal.pone.0165078)
Supplement: S3 Table — (PDF) [file pone.0165078.s015.pdf]

SUPPLEMENTARY TABLE 3

Multi-Gaussian fit parameters of step size and rupture force distributions from force-ramp data in Fig. S5

Protein	Peak1	Peak2	Peak3
Step size	23±5.3 nm	36±9.7 nm	66±22 nm
Rupture force	9.3±2.1 pN	23±6.6 pN	-

* Error bars are standard deviation of the population distribution.
