# Supplementary material for: CENP-A and H3 Nucleosomes Display a Similar Stability to Force-Mediated Disassembly
Source: PLoS One. 2016 Nov 7;11(11):e0165078. doi: 10.1371/journal.pone.0165078 (PMC5098787; doi:10.1371/journal.pone.0165078)
Supplement: S4 Table — (PDF) [file pone.0165078.s016.pdf]

SUPPLEMENTARY TABLE 4

Multi-Gaussian fit parameters of step size distribution from force-ramp data in Fig. 3c-d and S8 Fig.

Force (pN)	Protein	DNA	Peak1 (nm)	Peak2 (nm)	Peak3 (nm)
20	H3	Random	13±1.8	22±2.9	42±6.2
20	H3	Centromeric	13±0.9	22±3.6	47±7.1
20	CA	Random	13±1.9	22±3.0	42±4.9
20	CA	Centromeric	14±0.5	21±2.8	37±6.6
30	H3	Random	12±1.8	23±2.5	34±4.5
30	H3	Centromeric	13±1.7	20±3.0	35±4.4
30	CA	Random	15±1.1	21±3.5	39±4.8
30	CA	Centromeric	13±2.1	21±2.7	36±4.8
40	H3*	Random	-	18±3.7	35±3.9
40	H3	Centromeric	13±1.0	19±2.9	39±5.2
40	CA	Random	14±1.5	20±3.0	40±4.9
40	CA*	Centromeric	-	21±3.6	46±7.5

* Three-Gaussian peak fitting does not reduce chi-square value comparing to two-Gaussian peak fitting.

** Error bars are standard deviation of the population distribution.
