# Supplementary material for: CENP-A and H3 Nucleosomes Display a Similar Stability to Force-Mediated Disassembly
Source: PLoS One. 2016 Nov 7;11(11):e0165078. doi: 10.1371/journal.pone.0165078 (PMC5098787; doi:10.1371/journal.pone.0165078)
Supplement: S5 Table — (PDF) [file pone.0165078.s017.pdf]

SUPPLEMENTARY TABLE 5

Single-exponential fit parameters of life-time distribution from force-clamp data in Fig. 3e-g and S10 Fig.

Protein	DNA	20pN	30pN	40pN
H3	Random	77±6.1	32±2.9	14±2.1
H3	Centromeric	30±4.8	19±3.5	14±3.6
CA	Random	31±18	25±2.7	22±6.4
CA	Centromeric	41±6.1	35±2.9	8±3.0

* Error bars are standard deviation of the population distribution.
