# Supplementary material for: CENP-A and H3 Nucleosomes Display a Similar Stability to Force-Mediated Disassembly
Source: PLoS One. 2016 Nov 7;11(11):e0165078. doi: 10.1371/journal.pone.0165078 (PMC5098787; doi:10.1371/journal.pone.0165078)
Supplement: S1 Text — (PDF) [file pone.0165078.s018.pdf]

SUPPLEMENTARY TEXT 1

Sequence of the 7.9kb random DNA (randDNA)

(Ligation site for the biotin labeled DNA fragment)-
GGTCCACATGGTCTGCGGGTTCAGGGTGCACATCTGCCGCCAGGTCCGCGATGCCTGCGGGTTCAGGGTGTACGTC
AGCCGTCACTACTATGGGAGCCATTGGCTCGTAGATCGAGTCGATGTCACACGACTGCAAACTGGGTAGCGACAT
GGTATCGCTGGCAGTGGAAGACGAGTCGGTGGTCCAGTCCAAGTCAAACCCTCGTAACGACCGATCTGAGTGGTC
CGTCGTAGATGCGGCATACTTCCGCGGACTAACCACTGAAGGTACCGTCGGGTCGAACAGGAGAACCTTCTCGCA
CTTTACCTTCTGCACCCCATCTACATGGTATTTCGGGAGGGGAAAAGATGAGCAAACCACCATGCTTTTAACTTGG
TGTGACCTAAGGCGGGCGATCCGTTCTGCTGTCATTGCGTAGCGGCACAGGCAGGGCACTGTCCTGGGAGGTGTTG
ATGAATCGGAATCGTTCACCGGACATTTGGATCTGATGTTGTCCATTGTTTCGCCCAGCGCGTATAGGCATATCTGT
TCGTTTGCCTCTTGCAGTCTGGGCCACAACGTCAGTATCTCTGCCATATCAATAGCAGCCTGGTTGAATTTCGTACC
TTCAAAGTACGAGTACAGCGACCCGTCAGTGGTACTGTAGCCCTTACGACCCACCAGGCTGCTGTCCGGGTGCACT
CTCACCAAGTCTGTGGTCAGCTCCACGTCATCATTGAGCAACTCCACAGCCGTCCTCATGTCAATGGCTTCCTGGAT
TTTCTTCTCCCAACTTTTGTCTCTGCAGTAGATGGTCACGTCAGCGTCCGTGGCGTCCATTGCTGTGAATAGATGGT
TGAGGGATTGCTGCAGCCTATCTCTTCCGCCGCTGAACACTCCTGTGGACAGCAGCGGGATGGCTACGCTGCTCAG,
.TGACAGTCTGTTTACTTCGGCGGCCACTGCCCGGTAGACAGCGGCCAATTCGCGGTCCCCTTCCGCTTCAGTCGTG
GCAGAGAAATTAGGCGCTACAGCGTGGATGACGGGGTACGAGCCGCACATGACTGTTTTAATTGTGCCCACTGGT
GTTGCTGCTCCCTTAAAGGCTGACGGCCATTTCTTCGCCACGGCCCTGCATACGCCATCCCCTACAGTTCCACGGGC
GTTAGCTGCGTTAACCACAGCCGCTTCTGTGCACGTGGCTATGTCTGCTCTCTTAACTCTGTAGGATGGTGCACACC
CGGCCGTGTGCATGGCTTCTCCGGCATACACGGCACTCAGCTTGGTATTCATCTGGTGTAGCGTAGAGGGTCTCTTT
CCGTTGTCAAAGTTGGAGAACAGCAAGAACACTTCTGTATTGCTGGTGACACAATCCGGGCGCAACACTCTTGCAG
ACGAGAACTTTCTGCTTAAGGAGGAAACAACGGCTTCGCTGATTTTATCGGCGTATCCGTAAGCTCTCATCAAGAT
GCCGCCGGGTTTTAGCAGTCGTAGCGCATCTCCCCCAAGCATCTGCAGCTTCATGGCGTGGTCGACACACTGCTGG
TAGTGGTGGATTCTGAATTCCGTGTGAATGTTCACAAAGACCAAGTCGAACCTGCCGGCGTCAGCCGGCAGTCCTA
AACTTAGGTCGTAGCACCTATCGGCGCCTGTGACATTCAGCGGTGACAACCAAGTGACCCTGCGTCGAGGCAAAG
CCAGGTTGTACTCACTCACCAGCAGGACGTGGTACCCTCTTACTTTATTGACCAGCCACTCAACCCTACTGCCTTTA
ACCGTCTTGTACTCAGCCACCAGGGCGTGCGGCAGCCTGCGGTTGATAGGAATTACATTGTCCAGCACAGAAAGC
GGTTGGATTTTTCTTTCTGCGATAACTGCCTGCTTGCCCGTATGCCACTGCCCCTTCAGGAAGGTATGTCTAGCTTC
CAGCCTGGCAGCTGTTGCGGCATTGAATCCATACATCCTTCCACCAGGTCTGTTATCCCAGTGGTTGTTCTCGTAAT
ACAGGGACACCTTCGGGGCAGAAAACAGGCCACTGTCCAGGTCAACTCCATAGTACTTGGTGCAAATTTCATTCAA
GGCCACCACTGGAGAGTAAGCTCTGTCCTCCTTAAATGCTGTAATTATGGTGCTCCACTCCTCTGCTGTCAATCTGA
TTCCGGCAGTGTCCAGGACAGGCACCAGGCTTTTCGCCCAACACACGTTCGCTTTGTTCTGGAACGCGTCCACAGG
CGCAGCCGGTCCTTCAATCACCTTCATTATTTTGTCGTGTTCTTCTTGCCATTCTTCCAATGTGGCCGTAAAGTTACC
CTGTGGAATGTTTGATAGGACCTTAATCCAGGGATCGCCGGCCAGCGTTTTCCACACCAGCCTATCCTCAGTGCGC
GTCAGCAGTACATTCACGTGCTCCGACGCAGGGGCATACAAGGGATTTTCATTCACCTTCTGCCTTACGGCGTATA
CCCCTTTGCGGGTGAGGCCCTGAGATGCTGCTGCTGTCATGACTTCGTGTCCACGGTAGTCCAACTGCAGCTGCTTT
GCCCAGCCTCGGAAGCATGTTAACACGATGTCTCCTGGCTTGGGCTTGGTCTGTCCTGTGGTGTCTATGATTATGGG
TTTGTTGCACGGGTTGGTCGTGCGCATCTTGCCTCCGTAGTGCAACGTAGACACGATGGCCGTGACTGGACGCGTG

CAACGTCTGGATATACTTTTATGACATACTTCAGTGCAGATGTTGTGGTTGAAGTTCACCTTAAGCTGCATCATATT
GAAGAATCCGCATTGCTTGGGGTCTCCGCATAACACCACTTTGCTCCGAGGTTTAACAAGAGCAATTAGGGCCAGC
AGAGTACCGGAATGGCAAGCGAAAGCCTCGTCCACATATAGGATGTCCACGGCACGACGACACCCGTTTAGCAGG
ATGGAGTCACTGTTTTCCCTACTTGTCCCCTTCCCGCGGTGCTTCTTCACGTCGTTAACTATTTCCTGGCAGTTCTCC
TTCTTGCCGCTGGTGACCAGATCGTGTTTGGTCACGAGGCTCTTAATAATAGCAGACTTGCCTGATCCCGGAACCC
CAAAGACTCCTACTACTGTAGTCTTATATGGTGCCGACGGCCTGATCTTCAGCCCTTCGTAGGCGAATTCATGGAA
CGGGGGGTTGGTTAGCTCTCCCACCAACACCAAACCCGACGCTTCCTCTCTCTTGACGCAGCATTTTTTATCTACGT
CGAACACGTACTCGGCGTCAGTTCTTTCAGCTCTGACTTTCTCGTAGTTCTCCTCGTCGGTGTTCAGCGACGGTCCG
TGAACGGCAATATGGTATAGTTTCCTGTTGACGAACTCCCTTTCGTTGTACACCATAGTGGCGCTCTCGCTCAAAGC
TTGATATCGAATTCGGTGACCCTTACGCGAATCCGCTTTCAGACGTTGACTGGTCGCGTCTGGCAAAAGTTAAAGA
CCTGACGCCCGGCGAACTGACCGCTGAGTCCTATGACGACAGCTATCTCGATGATGAAGATGCAGACTGGACTGC
GACCGGGCAGGGGCAGAAATCTGCCGGAGATACCAGCTTCACGCTGGCGTGGATGCCCGGAGAGCAGGGGCAGC
AGGCGCTGCTGGCGTGGTTTAATGAAGGCGATACCCGTGCCTATAAAATCCGCTTCCCGAACGGCACGGTCGATGT
GTTCCGTGGCTGGGTCAGCAGTATCGGTAAGGCGGTGACGGCGAAGGAAGTGATCACCCGCACGGTGAAAGTCAC
CAATGTGGGACGTCCGTCGATGGCAGAAGATCGCAGCACGGTAACAGCGGCAACCGGCATGACCGTGACGCCTGC
CAGCACCTCGGTGGTGAAAGGGCAGAGCACCACGCTGACCGTGGCCTTCCAGCCGGAGGGCGTAACCGACAAGA
GCTTTCGTGCGGTGTCTGCGGATAAAACAAAAGCCACCGTGTCGGTCAGTGGTATGACCATCACCGTGAACGGCGT
TGCTGCAGGCAAGGTCAACATTCCGGTTGTATCCGGTAATGGTGAGTTTGCTGCGGTTGCAGAAATTACCGTCACC
GCCAGTTAATCCGGAGAGTCAGCGATGTTCCTGAAAACCGAATCATTTGAACATAACGGTGTGACCGTCACGCTTT
CTGAACTGTCAGCCCTGCAGCGCATTGAGCATCTCGCCCTGATGAAACGGCAGGCAGAACAGGCGGAGTCAGACA
GCAACCGGAAGTTTACTGTGGAAGACGCCATCAGAACCGGCGCGTTTCTGGTGGCGATGTCCCTGTGGCATAACCA
TCCGCAGAAGACGCAGATGCCGTCCATGAATGAAGCCGTTAAACAGATTGAGCAGGAAGTGCTTACCACCTGGCC
CACGGAGGCAATTTCTCATGCTGAAAACGTGGTGTACCGGCTGTCTGGTATGTATGAGTTTGTGGTGAATAATGCC
CCTGAACAGACAGAGGACGCCGGGCCCGCAGAGCCTGTTTCTGCGGGAAAGTGTTCGACGGTGAGCTGAGTTTTG
CCCTGAAACTGGCGCGTGAGATGGGGCGACCCGACTGGCGTGCCATGCTTGCCGGGATGTCATCCACGGAGTATG
CCGACTGGCACCGCTTTTACAGTACCCATTATTTTCATGATGTTCTGCTGGATATGCACTTTTCCGGGCTGACGTAC
ACCGTGCTCAGCCTGTTTTTCAGCGATCCGGATATGCATCCGCTGGATTTCAGTCTGCTGAACCGGCGCGAGGCTG
ACGAAGAGCCTGAAGATGATGTGCTGATGCAGAAAGCGGCAGGGCTTGCCGGAGGTGTCCGCTTTGGCCCGGACG
GGAATGAAGTTATCCCCGCTTCCCCGGATGTGGCGGACATGACGGAGGATGACGTAATGCTGATGACAGTATCAG
AAGGGATCGCAGGAGGAGTCCGGTATGGCTGAACCGGTAGGCGATCTGGTCGTTGATTTGAGTCTGGATGCGGCC
AGATTTGACGAGCAGATGGCCAGAGTCAGGCGTCATTTTTCTGGTACGGAAAGTGATGCGAAAAAAACAGCGGCA
GTCGTTGAACAGTCGCTGAGCCGACAGGCGCTGGCTGCACAGAAAGCGGGGATTTCCGTCGGGCAGTATAAAGCC
GCCATGCGTATGCTGCCTGCACAGTTCACCGACGTGGCCACGCAGCTTGCAGGCGGGCAAAGTCCGTGGCTGATCC
TGCTGCAACAGGGGGGGCAGGTGAAGGACTCCTTCGGCGGGATGATCCCCATGTTCAGGGGGCTTGCCGGTGCGA
TCACCCTGCCGATGGTGGGGGCCACCTCGCTGGCGGTGGCGACCGGTGCGCTGGCGTATGCCTGGTATCAGGGCA
ACTCAACCCTGTCCGATTTCAACAAAACGCTGGTCCTTTCCGGCAATCAGGCGGGACTGACGGCAGATCGTATGCT
GGTCCTGTCCAGAGCCGGGCAGGCGGCAGGGCTGACGTTTAACCAGACCAGCGAGTCACTCAGCGCACTGGTTAA
GGCGGGGGTAAGCGGTGAGGCTCAGATTGCGTCCATCAGCCAGAGTGTGGCGCGTTTCTCCTCTGCATCCGGCGTG
GAGGTGGACAAGGTCGCTGAAGCCTCTAGAAGTTCAGGAAGCGGTGATGCTGATAGAAGCCGGACTGAGTACCTA
CGAGAAAGAGTGCGCAAAACGCGGTGACGACTATCAGGAAATTTTTGCCCAGCAGGTCCGTGAAACGATGGAGCG
CCGTGCAGCCGGTCTTAAACCGCCCGCCTGGGCGGCTGCAGCATTTGAATCCGGGCTGCGACAATCAACAGAGGA

GGAGAAGAGTGACAGCAGAGCTGCGTAATCTCCCGCATATTGCCAGCATGGCCTTTAATGAGCCGCTGATGCTTGA
ACCCGCCTATGCGCGGGTTTTCTTTTGTGCGCTTGCAGGCCAGCTTGGGATCAGCAGCCTGACGGATGCGGTGTCC
GGCGACAGCCTGACTGCCCAGGAGGCACTCGCGACGCTGGCATTATCCGGTGATGATGACGGACCACGACAGGCC
CGCAGTTATCAGGTCATGAACGGCATCGCCGTGCTGCCGGTGTCCGGCACGCTGGTCAGCCGGACGCGGGCGCTG
CAGCCGTACTCGGGGATGACCGGTTACAACGGCATTATCGCCCGTCTGCAACAGGCTGCCAGCGATCCGATGGTG
GACGGCATTCTGCTCGATATGGACACGCCCGGCGGGATGGTGGCGGGGGCATTTGACTGCGCTGACATCATCGCC
CGTGTGCGTGACATAAAACCGGTATGGGCGCTTGCCAACGACATGAACTGCAGTGCAGGTCAGTTGCTTGCCAGTG
CCGCCTCCCGGCGTCTGGTCACGCAGACCGCCCGGACAGGCTCCATCGGCGTCATGATGGCTCACAGTAATTACGG
TGCTGCGCTGGAGAAACAGGGTGTGGAAATCACGCTGATTTACAGCGGCAGCCATAAGGTGGATGGCAACCCCTA
CAGCCATCTTCCGGATGACGTCCGGGAGACACTGCAGTCCCGGATGGACGCAACCCGCCAGATGTTTGCGCAGAA
GGTGTCGGCATATACCGGCCTGTCCGTGCAGGTTGTGCTGGATACCGAGGCTGCAGTGTACAGCGGTCAGGAGGC
CATTGATGCCGGACTGGCTGATGAACTTGTTAACAGCACCGATGCGATCACCGTCATGCGTGATGCACTGGATGCA
CGTAAATCCCGTCTCTCAGGAGGGCGAATGACCAAAGAGACTCAATCAACAACTGTTTCAGCCACTGCTTCGCAG
GCTGACGTTACTGACGTGGTGCCAGCGACGGAGGGCGAGAACGCCAGCGCGGCGCAGCCGGACGTGAACGCGCA
GATCACCGCAGCGGTTGCGGCAGAAAACAGCCGCATTATGGGGATCCTCAACTGTGAGGAGGCTCACGGACGCGA
AGAACAGGCACGCGTGCTGGCAGAAACCCCCGGTATGACCGTGAAAACGGCCCGCCGCATTCTGGCCGCAGCACC
ACAGAGTGCACAGGCGCGCAGTGACACTGCGCTGGATCGTCTGATGCAGGGGGCACCGGCACCGCTGGCTGCAGG
TAACCCGGCATCTGATGCCGTTAACGATTTGCTGAACACACCAGTGTAAGGGATGTTTATGACGAGCAAAGAAAC
CTTTACCCATTACCAGCCGCAGGGCAACAGTGACCCGGCTCATACCGCAACCGCGCCCGGCGGATTGAGTGCGAA
AGCGCCTGCAATGACCCCGCTGATGCTGGACACCTCCAGCCGTAAGCTGGTTGCGTGGGATGGCACCACCGACGG
TGCTGCCGTTGGCATTCTTGCGGTTGCTGCTGACCAGACCAGCACCACGCTGACGTTCTACAAGTCCGGCACGTTC
CGTTATGAGGATGTGCTCTGGCCGGAGGCTGCCAGCGACGAGACGAAAAAACGGACCGCGTTTGCCGGAACGGCA
ATCAGCATCGTTTAACTTTACCCTTCATCACTAAAGGCCGCCTGTGCGGCTTTTTTTACGGGATTTTTTTATGTCGAT
GTACACAACCGCCCAAGAGCTCCAGCTTTTGTTCCCTTTAGTGAGGGTTAATTGCGCGCTTGGCGTAATCATGGTC
ATAGCTGTTTCCTGTGTGAAATTGTTATCCGCTCACAATTCCACACAACATACGAGCCGGAAGCATAAAGTGTAAA
GCCTGGGGTGCCTAATGAGTGAGCTAACTCACATTAATTGCGTTGCGCTCACTGCCCGCTTTCCAGTCGGGAAACC
TGTCGTGCCAGCTGCATTAATGAATCGGCCAACGCGCGGGGAGAGGCGGTTTGCGTATTGGGCGCTCTTCCGCTTC
CTCGCTCACTGACTCGCTGCGCTCGGTCGTTCGGCTGCGGCGAGCGGTATCAGCTCACTCAAAGGCGGTAATACGG
TTATCCACAGAATCAGGGGATAACGCAGGAAAGAA-(Ligation site for the 500 bp-long DBCO labeled DNA)

Sequence of the centromeric DNA (cenDNA)

(Ligation site for the biotin labeled DNA fragment)-
GTGCTGGAATTCGCCCTTCATCACAAAGAAGTTTCTGAGAATGCTTCTGTCTAGTTTTTATACGAAGATATTCCCTT
TTCTGCCTTTGACCCCAAAGCGCTTGAAATCTCCACTTGCAAATTCCACAAAAACAGTGTTTCAAATCTGCTCTCTC
TAAATGAAAGTTCAACTCTGTCAGTTGAATACACTCAACACAAGGAAGTTACTGAGAATTCTTCTGTCTAGCATAA
TATGAAGAAATCCCGTTTCCAACGAAGGCCTCAAAGGGGTCTGAATATCCACTTGCAGACTTTATAAACAGAGTGT
TTACTAACTGCTCTATGAAAAGAAACGTTAAACTCTGTGAGTTGAACACACACATCACAAAGGAGTTTCTGAGAAT
CATTCTGTCTAGTTTTTCTACGAAGATATTTCCTTTTCTACTATTGACCTGAAAGCAGCTGAAATCTCCACTTGCAA

ATTCCACAAAAAGAGTGTTTCAAGTCTGCTCTGTGTAAAGGATCGTTCAACTCTGTGAGTTGAATACACACAACAC
AAGGAAGTTACTGAGAATTCTTCTCTCTAGCAGAATATGAAGAAATCCCGTTTCCAACGAAGGCCTCAAAGAGGTC
TGAATATCCACTTGCAGACTTTACAAACAGAGTGTTTCCTAACTGCTCTATGAAAAGGAAGGTTCAACTCTGTGAG
TTGAATGCAAAGGGCGAATTCTGCAGACGCTGTGCTGGAATTCGCCCTTCATCACAAAGAAGTTTCTGAGAATGCT
TCTGTCTAGTTTTTATACGAAGATATTCCCTTTTCTGCCTTTGACCCCAAAGCGCTTGAAATCTCCACTTGCAAATTC
CACAAAAACAGTGTTTCAAATCTGCTCTCTCTAAATGAAAGTTCAACTCTGTCAGTTGAATACACTCAACACAAGG
AAGTTACTGAGAATTCTTCTGTCTAGCATAATATGAAGAAATCCCGTTTCCAACGAAGGCCTCAAAGGGGTCTGAA
TATCCACTTGCAGACTTTATAAACAGAGTGTTTACTAACTGCTCTATGAAAAGAAACGTTAAACTCTGTGAGTTGA
ACACACACATCACAAAGGAGTTTCTGAGAATCATTCTGTCTAGTTTTTCTACGAAGATATTTCCTTTTCTACTATTG
ACCTGAAAGCAGCTGAAATCTCCACTTGCAAATTCCACAAAAAGAGTGTTTCAAGTCTGCTCTGTGTAAAGGATCG
TTCAACTCTGTGAGTTGAATACACACAACACAAGGAAGTTACTGAGAATTCTTCTCTCTAGCAGAATATGAAGAAA
TCCCGTTTCCAACGAAGGCCTCAAAGAGGTCTGAATATCCACTTGCAGACTTTACAAACAGAGTGTTTCCTAACTG
CTCTATGAAAAGGAAGGTTCAACTCTGTGAGTTGAATGCAAAGGGCGAATTCTGCAGAGCGTGTGCTGGAATTCG
CCCTTCATCACAAAGAAGTTTCTGAGAATGCTTCTGTCTAGTTTTTATACGAAGATATTCCCTTTTCTGCCTTTGACC
CCAAAGCGCTTGAAATCTCCACTTGCAAATTCCACAAAAACAGTGTTTCAAATCTGCTCTCTCTAAATGAAAGTTC
AACTCTGTCAGTTGAATACACTCAACACAAGGAAGTTACTGAGAATTCTTCTGTCTAGCATAATATGAAGAAATCC
CGTTTCCAACGAAGGCCTCAAAGGGGTCTGAATATCCACTTGCAGACTTTATAAACAGAGTGTTTACTAACTGCTC
TATGAAAAGAAACGTTAAACTCTGTGAGTTGAACACACACATCACAAAGGAGTTTCTGAGAATCATTCTGTCTAGT
TTTTCTACGAAGATATTTCCTTTTCTACTATTGACCTGAAAGCAGCTGAAATCTCCACTTGCAAATTCCACAAAAAG
AGTGTTTCAAGTCTGCTCTGTGTAAAGGATCGTTCAACTCTGTGAGTTGAATACACACAACACAAGGAAGTTACTG
AGAATTCTTCTCTCTAGCAGAATATGAAGAAATCCCGTTTCCAACGAAGGCCTCAAAGAGGTCTGAATATCCACTT
GCAGACTTTACAAACAGAGTGTTTCCTAACTGCTCTATGAAAAGGAAGGTTCAACTCTGTGAGTTGAATGCAAAGG
GCGAATTCTGCAGAGTATGTGTGCTGGAATTCGCCCTTCATCACAAAGAAGTTTCTGAGAATGCTTCTGTCTAGTTT
TTATACGAAGATATTCCCTTTTCTGCCTTTGACCCCAAAGCGCTTGAAATCTCCACTTGCAAATTCCACAAAAACAG
TGTTTCAAATCTGCTCTCTCTAAATGAAAGTTCAACTCTGTCAGTTGAATACACTCAACACAAGGAAGTTACTGAG
AATTCTTCTGTCTAGCATAATATGAAGAAATCCCGTTTCCAACGAAGGCCTCAAAGGGGTCTGAATATCCACTTGC
AGACTTTATAAACAGAGTGTTTACTAACTGCTCTATGAAAAGAAACGTTAAACTCTGTGAGTTGAACACACACATC
ACAAAGGAGTTTCTGAGAATCATTCTGTCTAGTTTTTCTACGAAGATATTTCCTTTTCTACTATTGACCTGAAAGCA
GCTGAAATCTCCACTTGCAAATTCCACAAAAAGAGTGTTTCAAGTCTGCTCTGTGTAAAGGATCGTTCAACTCTGT
GAGTTGAATACACACAACACAAGGAAGTTACTGAGAATTCTTCTCTCTAGCAGAATATGAAGAAATCCCGTTTCCA
ACGAAGGCCTCAAAGAGGTCTGAATATCCACTTGCAGACTTTACAAACAGAGTGTTTCCTAACTGCTCTATGAAAA
GGAAGGTTCAACTCTGTGAGTTGAATGCAAAGGGCGAATTCTGCAGATTGAGTGTGCTGGAATTCGCCCTTCATCA
CAAAGAAGTTTCTGAGAATGCTTCTGTCTAGTTTTTATACGAAGATATTCCCTTTTCTGCCTTTGACCCCAAAGCGC
TTGAAATCTCCACTTGCAAATTCCACAAAAACAGTGTTTCAAATCTGCTCTCTCTAAATGAAAGTTCAACTCTGTCA
GTTGAATACACTCAACACAAGGAAGTTACTGAGAATTCTTCTGTCTAGCATAATATGAAGAAATCCCGTTTCCAAC
GAAGGCCTCAAAGGGGTCTGAATATCCACTTGCAGACTTTATAAACAGAGTGTTTACTAACTGCTCTATGAAAAGA
AACGTTAAACTCTGTGAGTTGAACACACACATCACAAAGGAGTTTCTGAGAATCATTCTGTCTAGTTTTTCTACGA
AGATATTTCCTTTTCTACTATTGACCTGAAAGCAGCTGAAATCTCCACTTGCAAATTCCACAAAAAGAGTGTTTCAA
GTCTGCTCTGTGTAAAGGATCGTTCAACTCTGTGAGTTGAATACACACAACACAAGGAAGTTACTGAGAATTCTTC
TCTCTAGCAGAATATGAAGAAATCCCGTTTCCAACGAAGGCCTCAAAGAGGTCTGAATATCCACTTGCAGACTTTA

CAAACAGAGTGTTTCCTAACTGCTCTATGAAAAGGAAGGTTCAACTCTGTGAGTTGAATGCAAAGGGCGAATTCTG
CAGA-(Ligation site for the DBCO labeled DNA fragments)

Sequence of the biotin-labeled DNA fragment

(This fragment is made by PCR amplification with biotin-dUTP)

GACCGAGATAGGGTTGAGTGTTGTTCCAGTTTGGAACAAGAGTCCACTATTAAAGAACGTGGACTCCAACGTCAA
AGGGCGAAAAACCGTCTATCAGGGCGATGGCCCACTACGTGAACCATCACCCTAATCAAGTTTTTTGGGGTCGAG
GTGCCGTAAAGCACTAAATCGGAACCCTAAAGGGAGCCCCCGATTTAGAGCTTGACGGGGAAAGCCGGCGAACGT
GGCGAGAAAGGAAGGGAAGAAAGCGAAAGGAGCGGGCGCTAGGGCGCTGGCAAGTGTAGCGGTCACGCTGCGCG
TAACCACCACACCCGCCGCGCTTAATGCGCCGCTACAGGGCGCGTCCCATTCGCCATTCAGGCTGCGCAACTGTTG
GGAAGGGCGATCGGTGCGGGCCTCTTCGCTATTACGCCAGCTGGCGAAAGGGGGATGTGCTGCAAGGCGATTAAG
TTGGGTAACGCCAGCTGG (Ligation site for the random/cenDNA fragment)

Sequence of the DBCO-labeled DNA fragment

(This fragment is made by PCR amplification with DBCO-dUTP)

(Ligation site for the random/cenDNA fragment)-
CCTACTGGCGTTACCCAACTTAATCGCCTTGCAGCACATCCCCCTTTCGCCAGCTGGCGTAATAGCGAAGAGGCCC
GCACCGATCGCCCTTCCCAACAGTTGCGCAGCCTGAATGGCGAATGGGACGCGCCCTGTAGCGGCGCATTAAGCG
CGGCGGGTGTGGTGGTTACGCGCAGCGTGACCGCTACACTTGCCAGCGCCCTAGCGCCCGCTCCTTTCGCTTTCTTC
CCTTCCTTTCTCGCCACGTTCGCCGGCTTTCCCCGTCAAGCTCTAAATCGGGGGCTCCCTTTAGGGTTCCGATTTAG
TGCTTTACGGCACCTCGACCCCAAAAAACTTGATTAGGGTGATGGTTCACGTAGTGGGCCATCGCCCTGATAGACG
GTTTTTCGCCCTTTGACGTTGGAGTCCACGTTCTTTAATAGTGGACTCTTGTTCCAAACTGGAACAACACTCAACCC
TATCTCGGTC
