# Supplementary material for: CENP-A and H3 Nucleosomes Display a Similar Stability to Force-Mediated Disassembly
Source: PLoS One. 2016 Nov 7;11(11):e0165078. doi: 10.1371/journal.pone.0165078 (PMC5098787; doi:10.1371/journal.pone.0165078)
Supplement: S2 Text — (PDF) [file pone.0165078.s019.pdf]

SUPPLEMENTARY TEXT 2

Step-finding algorithm

To identify the positions and sizes of steps in the DNA-extension time trace and in the force-extension curve, we developed a step-finding algorithm. Due to the nonlinear nature of the force-extension curves, the algorithm considers only the local data points within a small window around each step. Considering the slow speed of the force increment (0.1 pN/s) and the corresponding DNA extension rate (typically ~20 nm/pN at 10 pN and ~5 nm/pN at 40 pN of the 3.5 and 7.9 kb DNA at our buffer condition), which are much slower than our sampling rate of the bead tracking (10~50 Hz), we set the window size to be 20~50 data points depending on the noise level of each trace, so that the force-induced DNA extension shift within a window is negligible. Within a window, the median of the first half of the data points were compared to that of the second half. If the difference was bigger than 3 times the noise level defined as the Allan deviation within the time window (1), we accepted the center data point as a step. Further selections of the identified steps were made based on the following criteria: 1) two steps should be separated by more than the half size of the window, and 2) the size of the step should be larger than 10nm. Finally, apparent false detections due to uncharacterized perturbations (such as mechanical vibration from environmental noise) were removed manually. Finally, the most likely trajectories were reconstructed from the identified steps as well as the mean values between them.

References

1. Allan, D.W. (1966) Statistics of Atomic Frequency Standards. *Pr Inst Electr Elect*, **54**, 221-230.
